# Extracellular calcium alters calcium-sensing receptor network integrating intracellular calcium-signaling and related key pathway

**DOI:** 10.1038/s41598-021-00067-2

**Published:** 2021-10-18

**Authors:** Rakshya Gorkhali, Li Tian, Bin Dong, Pritha Bagchi, Xiaonan Deng, Shrikant Pawar, Duc Duong, Ning Fang, Nicholas Seyfried, Jenny Yang

**Affiliations:** 1 Department of Chemistry, Center of Diagnostics and Therapeutics, Advanced Translational Imaging Facility, Georgia State University, Atlanta, GA 30303 USA; 2 Department of Biology, Center of Diagnostics and Therapeutics, Advanced Translational Imaging Facility, Georgia State University, Atlanta, GA 30303 USA; 3 Emory Integrated Proteomics Core, Emory University School of Medicine, Atlanta, GA 30322 USA; 4 Department of Biochemistry, Emory University School of Medicine, Atlanta, GA 30322 USA

**Keywords:** Proteomics, Calcium signalling

## Abstract

G-protein-coupled receptors (GPCRs) are a target for over 34% of current drugs. The calcium-sensing receptor (CaSR), a family C GPCR, regulates systemic calcium (Ca^2+^) homeostasis that is critical for many physiological, calciotropical, and noncalciotropical outcomes in multiple organs. However, the mechanisms by which extracellular Ca^2+^ (Ca^2+^_ex_) and the CaSR mediate networks of intracellular Ca^2+^-signaling and players involved throughout the life cycle of CaSR are largely unknown. Here we report the first CaSR protein–protein interactome with 94 novel putative and 8 previously published interactors using proteomics. Ca^2+^_ex_ promotes enrichment of 66% of the identified CaSR interactors, pertaining to Ca^2+^ dynamics, endocytosis, degradation, trafficking, and primarily to protein processing in the endoplasmic reticulum (ER). These enhanced ER-related processes are governed by Ca^2+^_ex_-activated CaSR which directly modulates ER-Ca^2+^ (Ca^2+^_ER_), as monitored by a novel ER targeted Ca^2+^-sensor. Moreover, we validated the Ca^2+^_ex_ dependent colocalizations and interactions of CaSR with ER-protein processing chaperone, 78-kDa glucose regulated protein (GRP78), and with trafficking-related protein. Live cell imaging results indicated that CaSR and vesicle-associated membrane protein-associated A (VAPA) are inter-dependent during Ca^2+^_ex_ induced enhancement of near-cell membrane expression. This study significantly extends the repertoire of the CaSR interactome and reveals likely novel players and pathways of CaSR participating in Ca^2+^_ER_ dynamics, agonist mediated ER-protein processing and surface expression.

## Introduction

Calcium-sensing receptor (CaSR) belongs to class-C of the largest cell surface receptor family, the G-protein-coupled-receptors (GPCR’s), which are targets to over 34% of the Food and Drug Administration (FDA) approved drugs in the United States^1^. CaSR helps maintains a tight systemic Ca^2+^ homeostasis between the extracellular space (10^−3^ M) and cytosol (10^−7^ to 10^−8^ M) to control numerous processes including cellular communication, secretion, apoptosis, chemotactic responses, cell proliferation, cytoskeletal rearrangements, ion channel activity, the control of gene expression, and cell differentiation^2–6^. Thus, homeostasis is critical for many (patho)physiological processes in multiple organs, including parathyroid, kidney, heart, bone, brain, and skin^7^. CaSR activated by extracellular Ca^2+^ (Ca^2+^_ex_) triggers multiple intracellular signaling pathways transduced through heterotrimeric G proteins; Gq/11, Gi/o, G12/13, and Gs^3,8–11^ (Fig. S1). Thereof, the CaSR-mediated Ca^2+^ signaling cascade regulates sub-cellular Ca^2+^ concentrations (Ca^2+^), including, Ca^2+^_cyt_, Ca^2+^_ER_ and mitochondrial Ca^2+^ (Ca^2+^_mito_)^12–14^. One of the major CaSR mediated Gq/11 transduced pathways involved the activation of phospholipase C (PLC), which in turn raises the cytosolic Ca^2+^ (Ca^2+^_cyt_) and results in Ca^2+^_cyt_ oscillation through inositol triphosphate (IP_3_) induced endoplasmic reticulum Ca^2+^ (Ca^2+^_ER_) release^12^.

This CaSR mediated crosstalk between the extra- and intra- cellular Ca^2+^ signaling is integral to protein biosynthesis and trafficking, including regulation of CaSR abundance, as well as inducing its active conformation and dictating its dynamic life cycle^15^. Prolonged exposure to Ca^2+^_ex_ is known to mobilize the intracellular pool of nascent CaSR from the ER^16^, Golgi, and ER-Golgi intermediate compartments (ERGIC) to the plasma membrane through anterograde transport^15^. CaSR is an exceptional receptor with minimal functional desensitization in the chronic presence of agonist^17^. Subsequently, CaSR undergoes retrograde transport to lysosome or proteasome for degradation^15^. Perturbation in protein biosynthesis and trafficking are observed in physiological disorders, including diabetes mellitus and vascular and neurological diseases that alter Ca^2+^ mediated signaling, such as in the ER^15^. Additionally, mutations in CaSR and its binding partners and subsequent dysfunctions in CaSR mediated Ca^2+^ signaling are closely associated with calciotropic (familial hypocalciuric hypercalcemia (FHH), neonatal severe hyperparathyroidism (NSHPT), autosomal dominant hypocalcemia (ADH), and secondary hyperparathyroidism) and noncalciotropic disorders (cancers, Alzheimer’s disease, pancreatitis, diabetes mellitus, hypertension and bone and gastrointestinal disorders)^18–20^.

To date, only a handful of CaSR interactors have been identified^15,21–31^. Proteins involved in anterograde trafficking of CaSR are small GTP binding proteins (Rabs^21,22^, Sar1^23,24^ and ARFs^25^), cargo/chaperones (p24A^23^, RAMPs^26^) and interacting proteins (14-3-3 proteins^15,27,28^, and CaM^32^). Further, CaSR endocytosis is facilitated by G protein receptor kinases (GRKs)^29^, protein kinase C^30^ and β-arrestins^29,31^. A recent study has shown an interaction between CaSR and AP2S1, and AP2S1 has been shown to facilitate CaSR endocytosis^33^. Finally, CaSR is degraded in the proteasome or lysosome following ubiquitination by E3 ubiquitin ligase, dorfin^34^. While the physiological significance of CaSR-mediated Ca^2+^ signaling and information on sparse CaSR-binding partners has been established, knowledge related to how Ca^2+^_ex_ and CaSR orchestrate Ca^2+^ dynamics and signaling as well how they harmonize key regulators for critical cellular processes is incomplete. This is due in part to limitations involved in studying membrane proteins, and challenges in capturing transient interactions^15,21–28,32^. In our study we have aimed to capture proteins that interact with CaSR throughout its life cycle from signaling, internalization, endocytosis and synthesis to insertion through agonist-derived insertional signaling (ADIS)^15^.

To address the above long-standing question, we have characterized the first CaSR-protein–protein interaction (CaSR-PPI) network using quantitative proteomics with tandem mass spectrometry (LC–MS/MS) coupled with Co-IP, with and without Ca^2+^_ex_ in HEK293 cells. Many previous CaSR related studies have been carried out in HEK293 cells^15,16,35^. We mapped 94 novel putative and 8 previously published CaSR interactors. Further, we revealed a distinct Ca^2+^_ex_ dependent enrichment in gene ontology annotations related to the ER. Moreover, we characterized Ca^2+^_ex_ mediated interactions of CaSR with two major regulatory proteins, vesicle-associated membrane protein-associated A (VAPA) involved in anterograde trafficking^36,37^ from the ER to the Golgi and 78-kDa glucose regulated protein (GRP78/Bip/HSPA5) involved in protein processing in the ER^38^. We have shown that CaSR and VAPA are inter-dependent of each other for the Ca^2+^_ex_ enhanced near-cell membrane expression in Cos7 cells. Thus, combining our study with known functions, we provide a larger repertoire of the CaSR interactome in HEK293 cells, and reveal likely novel players and pathways of CaSR participating in: Ca^2+^_ER_ dynamics; agonist mediated ER-protein processing, such as by GRP78; and surface expression via key regulators such as VAPA in different tissues. The established CaSR-PPI offers a novel paradigm for understanding the molecular bases of CaSR associated diseases and facilitating drug development.

## Results

### Capture of potential CaSR interactors in the presence of Ca^2+^_ex_ and EGTA

**Figure 1:**
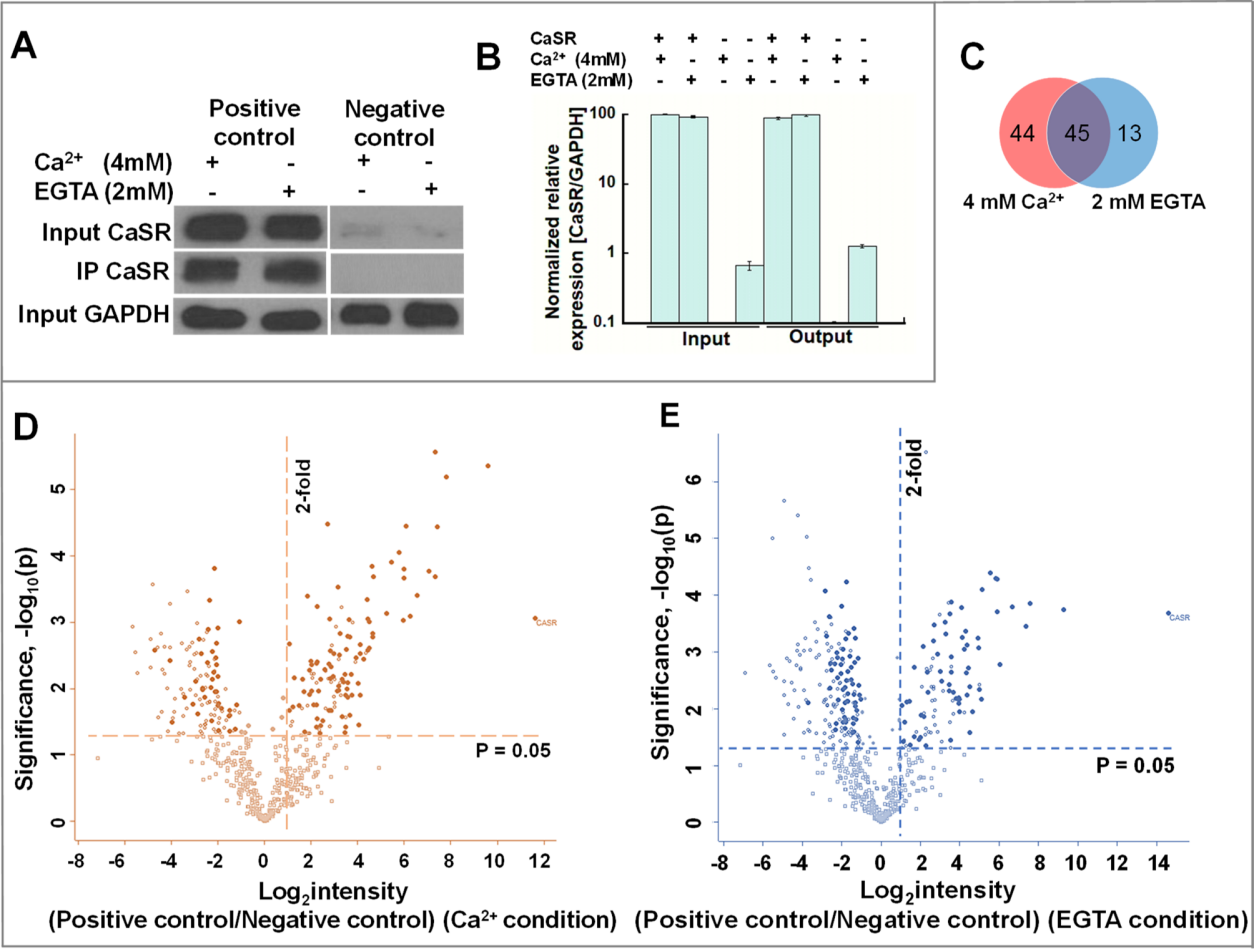
Isolation of potential CaSR interactors. (**A**) Co-immunoprecipitation experiments using anti-FLAG antibody on total cell extracts from HEK293 followed by western blot analysis with anti-CaSR antibody and anti-GAPDH (for total) was carried out in triplicates (one representative blot is shown). (**B**) Qualitative analysis of CaSR expression as normalized to the GAPDH in A (band intensity of CaSR divided by band intensity of GAPDH). (**C**) Venn diagram representing the number of proteins detected for each of the conditions. (**D,E**) Volcano plot of CaSR interactors (n = 3) enriched in 4 mM Ca^2+^ ((**D**), brown dots in right upper quadrant) and 2 mM EGTA perturbation (E, blue dots in upper right quadrant) in pairwise experiments versus negative control (without CaSR expression); n = 3. A total of 623 proteins are plotted (**D,E**) X-axis depicts enrichment in CaSR transfected (positive control) as compared to negative control. Y-axis depicts significance with p-value.

To identify proteins interacting with CaSR, we employed HEK293 cells transfected with either recombinant FLAG-tagged CaSR pcDNA3.1 (positive control) or empty pcDNA3.1 (negative control)^39–41^. Further, to establish the role of Ca^2+^_ex_ in interactions, the serum-starved cells were treated with either 4 mM Ca^2+^_ex_ (based on previously reported half maximal CaSR-activation [Ca^2+^])^42,43^ or 2 mM ethylene glycol-bis(β-aminoethyl ether)-N,N,N′,N′-tetra acetic acid (EGTA) (to chelate Ca^2+^_ex_), followed by subjection to immunoprecipitation with anti-FLAG antibody, LC–MS/MS (90% sample) and western-blot (10% sample) (Fig. S2). Serum starvation was carried out in order to synchronize the cellular activity in the cells to attain comparable responses throughout the cells. All experiments were run in triplicate and one representative blot is shown (Fig. 1A). CaSR was absent in negative controls. Between the Ca^2+^_ex_ and EGTA treatment conditions in the positive control, we detected little to no change in the amount of total CaSR input (Fig. 1A, left upper panel; Fig. 1B, first and second bar) or immunoprecipitated CaSR output (Fig. 1A, middle panels; Fig. 1B, fifth and sixth bar). This was validated by the MS intensities (averaged over three replicates) detected for CaSR outputs being < 1.25-fold different; in logarithmic (log2) scale, 33.13 in the presence of Ca^2+^_ex_ as compared to 32.83 in EGTA.

A total of 623 proteins (Supplemental Table S3) detected by LC–MS/MS were sorted and 106 were identified with high confidence as reliable CaSR interactors between the Ca^2+^_ex_ and EGTA treatment conditions (Supplemental Table S1). The following stringencies were employed to ensure robust upregulation, reproducibility, and detection**:** (i) treatments and IP for the positive and negative controls were performed in triplicates; (ii) identified proteins were at least twofold enriched in the CaSR transfected samples over the negative controls, i.e., log_2_ (HEK293 + CaSR-FLAG pcDNA/Hek293 + pcDNA3.1) ≥ 1.00; (iii) P-values were calculated using a two-sided Student’s t-test, with a null hypothesis that there is no difference in protein relative abundance between the two groups, and a two tailed t-test with P ≤ 0.05 between the groups being statistically significant; (iv) proteins had a minimum peptide spectrum match (PSM) of two for at least two replicates, and; (v) proteins were identified with at least one unique peptide. To compliment the study, LC–MS/MS was carried out on the total cell lysates to discern if the changes in enrichment after IP were due to the change in the total cellular expressions of these protein after Ca^2+^_ex_ or EGTA treatments. In all, 88% of the 106 proteins of interest displayed no significant change in the total protein expression (i.e., less than onefold change or [log_2_ intensity (Ca^2+^/EGTA) ≤ 0.58 and >  − 0.58]) (Fig. S3), implying true representation of CaSR-binding proteins. Besides these 93 true interactors, seven proteins that were not detected in the total lysates (MT-ATP8, WDR6, 54 HBB, TBL2, RALBP1 and GPALPP1) and two (PTPN1, TMCO1) that showed poor reproducibility between replicates, were also considered for further analysis (total of 102). Conversely, four proteins (HSPA6/7, IRS4, PTDSS1, SPTLC1) that had greater than onefold changes in protein expression data were removed from further studies.

Out of 102 CaSR interacting partners, eight previously known CaSR interactors involved in signaling and trafficking were re-validated: GNAI (Gα_i_)^39,44,45^, GNB2 (Gβ_2_)^39,44,45^, YWHAQ (14–3-3 θ)^28^, YWHAZ (14-3-3 ζ)^28^, YWHAE (14-3-3 ε)^28^, YWHAG (14-3-3 γ)^28^, TMED9 (p24)^23^ and CANX (calnexin)^15^. Additionally, 44 were exclusively identified in the presence of Ca^2+^_ex_, 13 exclusively in EGTA, and 45 were found common in both conditions (Fig. 1C). The volcano plots depict significantly enriched (log_2_ intensity [CaSR/pcDNA3.1) ≥ 1, -log_10_ (P) ≥ 1.3] potential CaSR interactors in the presence of Ca^2+^_ex_ (Fig. 1D, solid brown dots in upper right quadrant) and EGTA (Fig. 1E, solid blue dots in the upper right quadrant). Of these proteins, 88% were enriched by ≥ threefold (log_2_ intensity [i.e., CaSR/pcDNA3.1) ≥ 1.58]. CaSR had the highest log_2_ intensity (fold change) in both samples treated with either Ca^2+^_ex_ at 11.61 ± 1.07 [and −log_10_ (P) = 3.05] or EGTA at 14.32 ± 0.76 [and −log_10_(P) = 3.85].

### Ca^2+^_ex_ enriches putative CaSR interactors

**Figure 2:**
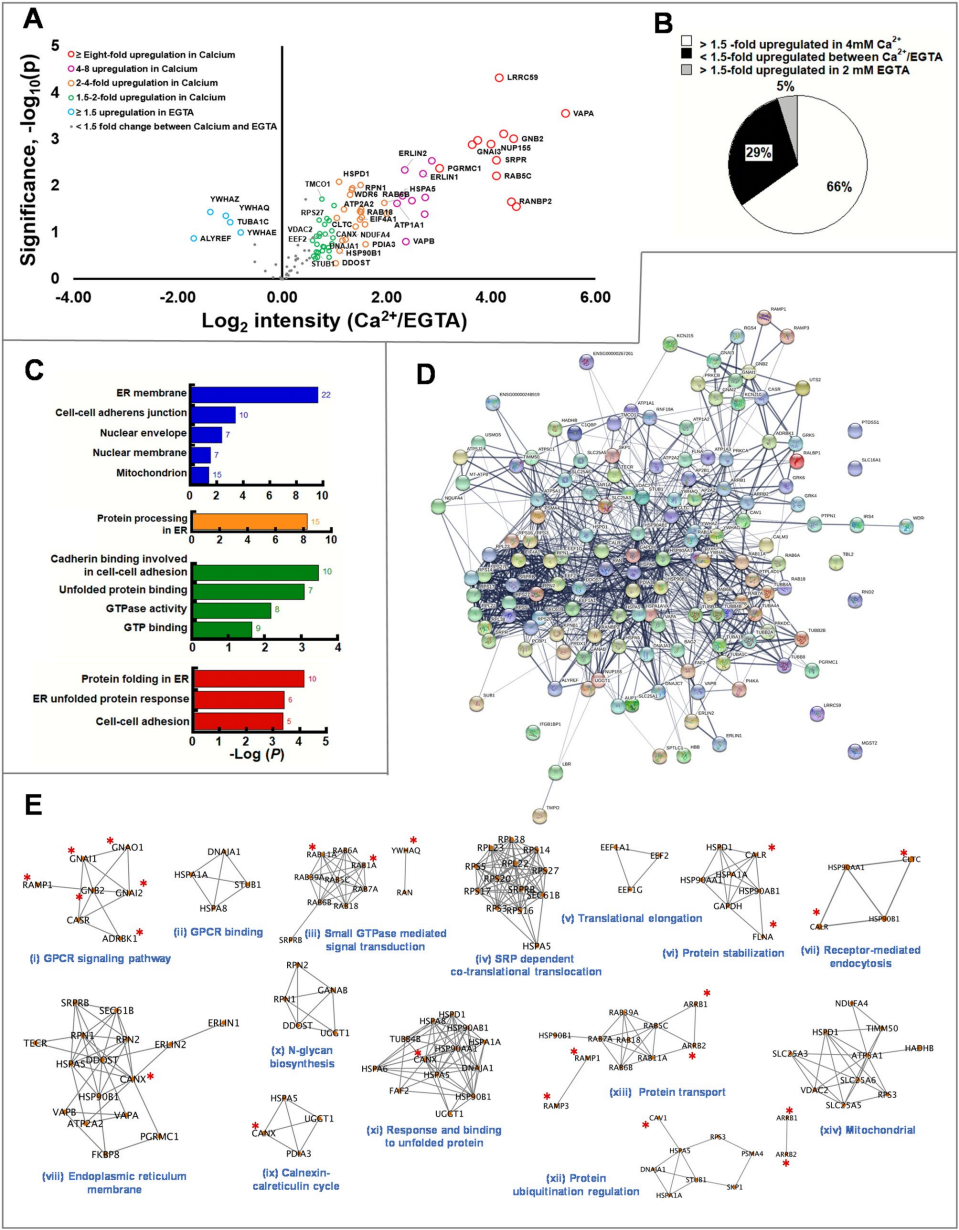
Extracellular Ca^2+^ promotes enrichment of CaSR-PPI in the ER. (**A**) Volcano plot of putative 102 CaSR interactors (n = 3) differentially up-regulated at various degrees in the presence of 4 mM [Ca^2+^] (right quadrant) and enriched in 2 mM EGTA (left quadrant). Red open circles represent ≥ eightfold [log_2_ intensity (fold change) =  ≥ 4]. Magenta open circles represent four to eightfold [log_2_ intensity (fold change) =  < 4 and ≥ 2]. Orange open circles represent two to fourfold [log_2_ intensity (fold change) < 2 and ≥ 1.5]. Green open circles represent 1.5–twofold [log_2_ intensity (fold change) < 1.5 ≥ 0.58]. Blue open circles represent down-regulation by ≥ 1.5-fold [log_2_ intensity (fold change) =  ≤  − 0.58]. Grey closed circles represent 29% detected with less than 1.5-folds change in abundance for the two conditions [log_2_ intensity (Ca^2+^/EGTA) =  < 0.58 and >  − 0.58]. The size of the circles represents the degree of up-regulation. (**B**) Venn diagram of the distribution of proteins differentially up-regulated in Ca^2+^. (**C**) Overrepresented gene-ontologies among the 66% CaSR interactors upregulated by ≥ 1.5-fold in presence of 4 mM Ca^2+^ examined with DAVID**.** Bonferroni-corrected P ≤ 0.05 and an enrichment ≥ 1.3 were used as a cut-off. The numbers on top of each bars represent the number of genes involved. Representation of the enrichment annotation for cellular compartment (blue bar), KEGG pathway (orange bar)^49–51^, molecular function (green bar), and biological processes (red bar). (**D**) A visual representation PPI for putative 102 CaSR interactors and 11 literature-curated CaSR-interactors generated using STRING. Spheres or nodes of different colors represent proteins. Large nodes represent proteins with known 3D structure while small nodes represent proteins with unknown structure. The lines between the nodes represent “edges”. The thicker and darker the edge is, the higher the confidence score for a true interaction based on available evidence. The network edge confidence is ranked between 0 and 1, with 1 being the most confident score. The strength of connecting nodes is not represented by the length or the location of the nodes. (**E**) Diagram of subnetwork of network in D containing functional protein clusters related to GPCR signaling, protein processing, quality control, endocytosis, trafficking, and mitochondria. Subclusters of putative 102 CaSR interactors and 11 literature-curated CaSR interactors examined by DAVID and grouped to biologically relevant annotations, including cellular compartment, molecular function and biological processes, are constructed using Cytoscape. The proteins with red asterisk are proteins known in the literature to interact with CaSR.

To discern whether there is a Ca^2+^_ex_ dependent regulation in CaSR networks, the 102 putative CaSR interactors were further evaluated for their differential MS intensities between Ca^2+^_ex_ and EGTA treatment groups using log_2_ intensity of (Ca^2+^/EGTA) (Fig. 2A,B, Supplementary Fig. S7). Remarkably, in the presence of Ca^2+^_ex_, 66% of these proteins were significantly enriched at varying degrees from 2 to 43-fold (log_2_ intensity (Ca^2+^/EGTA) = 1.00–5.42) (Fig. 2A, right quadrant and Fig. 2B). Conversely, there were five interactors that were enriched in the presence of EGTA by ≥ 1.5-fold [log_2_ intensity (Ca^2+^/EGTA) =  ≤ − 0.58]. These proteins included 14-3-3-θ (YWHAQ), 14-3-3-ζ (YWHAZ), 14-3-3-ε (YWHAE), Tubulin α-1C (TUBA1C) and THO complex subunit 4 (ALYREF) (Fig. 2A, left quadrant and Fig. 2B). In 29% of the 102 proteins only insignificant changes were observed between the Ca^2+^_ex_ and EGTA treatments, i.e., < 1.5-fold change in abundance [log_2_ intensity (Ca^2+^/EGTA) ≤ 0.58 and >  − 0.58] (Fig. 2B). Proteins in this group were primarily ribosomal proteins, tubulins, and heat shock proteins.

### Establishment of PPI network of CaSR

We created the functional annotation clusters for Ca^2+^_ex_ enriched CaSR interactors, consisting of cellular compartment, molecular function, and biological process using Database for Annotation, Visualization, and Integrated Discovery (DAVID), version 6.8^46–48^ (Fig. 2C, Supplemental Table S2). The clusters of identified proteins were significantly enriched in the ER membrane with the most significant KEGG pathway^49–51^ being protein processing in the ER. The enriched molecular functions were: GTP binding, GTPase activity, unfolded protein binding and cadherin binding. Concomitantly, biological processes such as protein folding in the ER, ER-unfolded protein response and cell–cell adhesion were most significantly enriched. The 102 total putative CaSR interactors along with 11 literature curated CaSR-interactors were mapped using Search Tool for the Retrieval of Interacting Genes/Proteins (STRING)^52^. The CaSR-PPI scored a local clustering coefficient of 0.492 with 1062 edges and a PPI enrichment of P < 1.00E−16 with high confidence (Fig. 2D). The clustering coefficient is a measure of how connected the nodes in the network are: highly connected networks have high values.

**Figure 3:**
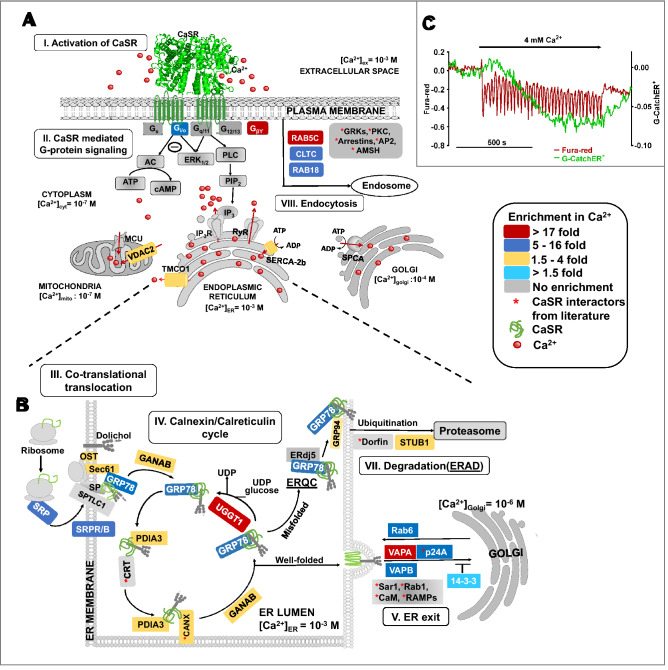
The schematic model of CaSR mediated signaling, biosynthesis and ER quality control of CaSR invoked through Ca^2+^_ext_ perturbation. Identified proteins from the experiment are colored respectively with fold-enrichment (log_2_(Ca^2+^/EGTA)-positive controls). (**A**) (I) CaSR is activated with 4 mM Ca^2+^_ex_. (II) Ca^2+^_ex_/CaSR mediated G-protein signaling through phospho-lipase C(PLC) activation and subsequently, IP_3_ invoked release of Ca^2+^ from the ER. Ca^2+^ dependent Gα_i_ invoked inhibition of acetyl cyclase is induced. (**B**) Alteration in Ca^2+^_ER_ induce amplification of CaSR interactomes. Biosynthesis and processing in the ER. (III) Co-translational translocation: The newly synthesized polypeptide bound to the SRP is directed to the ER membrane to the Sec complex by the SRP receptor, then processed by oligosaccharyl transferases (OST: DDOST and RPN1/2). GRP78 chaperones CaSR polypeptide to ER lumen where glucosidases GANAB allows for the removal of the outermost glucose residues. (IV) *Calnexin cycle* CANX and/or CRT along with GRP78 promotes folding and PDIA3 catalyzes disulfide formation. (V) *ER-exit* Properly folded CaSR traffics from ER to the Golgi assisted by VAPA and VAPB as well as p24A. Rab6 assists in retrograde trafficking from Golgi to the ER. 14-3-3 allows the CaSR to remain in the ER in the absence of Ca^2+^. UGGT acts as a check- point for improperly folded CaSR. [VI] Degradation pathway: Terminally misfolded CaSR undergoes ERAD and is ubiquitinated through STUB1. (VII) Endosomal degradation: CaSR from the plasma membrane can be endocytosed assisted by Rab5, Rab18 and CLTC. (**C**) Ca^2+^_ER_ release via CaSR monitored using G-CatchER^+^ (green line). Activated CaSR mediated Ca^2+^_i_ mobilized from ER measured using Fura-red (red line).

The established novel CaSR-PPI consisted of Ca^2+^_ex_ enriched sub-clusters, including key signaling G proteins GNAI (Gα_i_)^39,44,45^ and GNB2 (Gβ_2_)^39,44,45^ (Figs. 2E,(i), 3A,(II)), and intracellular Ca^2+^-handling proteins associated with Ca^2+^_ex_/CaSR mediated pathways in the ER (ATP2A2/SERCA-2b^53^ (Figs. 2E, (vii) and 3A, (II)) and TMCO1^54^ (Figs. 2E,(vii) and 3A,(II))) and the sarcoplasmic reticulum-mitochondrion interface (VDAC2)^55,56^, (Figs. 2E,(xiv) and 3A,(II)). In addition, the CaSR-PPI also delineated Ca^2+^_ex_ enriched sub-clusters related to co-translational translocation, or response to unfolded protein (ribosomal proteins, SRPR/B, SEC61, GRP78, DDOST, RPN1/2, GANAB)^57^ (Fig. 2E,(iv,x,xi) and 3B,(III)), calnexin cycle (GRP78, CANX, PDIA3^58^, UGGT1)^57^ (Figs. 2E,(ix,xi) and 3B,(IV)), ER/Golgi trafficking (RAB6^59^, VAPA^60,61^ and VAPB^39,44^) (Figs. 2E,(iii,viii,xiii), 3B, (V)), endocytosis (RAB18^62^, RAB5c^63,64^ and clathrin heavy chain (CLTC)^63^ (Figs. 2E,(iii, vii, xiii), 3A,(VIII)), and regulation of ubiquitin degradation pathway (STUB1/HSPA5^65^, RANBP2^65^, HSP90AA1/GRP94^57^, DNAJ1^66^, SKP1^67^) (Figs. 2E,(ii, iv, vi, vii, ix, xi, xii), 3B,(VII)). Our studies suggest a key integrational role of CaSR for extracellular- and multiple intra-organellar- Ca^2+^ signaling with major ER-protein processing, quality control, and trafficking pathways, and in mitochondrion transportation (TIMM50^68^, VDAC2, SLC25A, ATP5A1, HSPD1, NDUFA4, and PRDX1^55,56^, Figs. 2E,(xiv), 3A,(II), Supplemental Table S2).

### Ca^2+^_ex_ activated CaSR mediates Ca^2+^_ER_ release

To enhance understanding of the role of CaSR and Ca^2+^_ex_ in Ca^2+^_ER_ dynamics, we monitored rapid Ca^2+^_ER_ release using our recently designed ER sensor, G-CatchER^+^^69,70^. G-CatchER^+^ is a genetically encoded calcium sensor (GECI) designed with Ca^2+^ binding site on the surface of the beta barrel in a chromophore sensitive location of enhanced green fluorescent protein (EGFP)^70^. The inositol 1,4,5-triphosphate receptor (IP_3_R) is a Ca^2+^ release receptor, and sarco-endoplasmic reticulum calcium ATPase (SERCA) is a Ca^2+^ uptake receptor located on the membranes of the ER that regulate Ca^2+^_ER_ (Fig. S1). Activation of CaSR induces Ca^2+^_cyt_ oscillation resulting from Ca^2+^_ER_ release from IP_3_R as determined by Fura-red^12^ (Fig. 3C). We demonstrated direct evidence of Ca^2+^_ER_ release monitored by G-CatchER^+^ due to Ca^2+^_ex_ mediated CaSR activation (Fig. 3C). Therefore, with our proteomics, gene ontology and functional studies, we discovered the first implicit findings of the integration of the Ca^2+^_ER_, potential protein processing and trafficking units of the ER, and Ca^2+^ signaling mediated by Ca^2+^_ex_ -activated CaSR.

### Ca^2+^_ex_ enriches the interaction of CaSR with trafficking assistant protein VAPA

**Figure 4:**
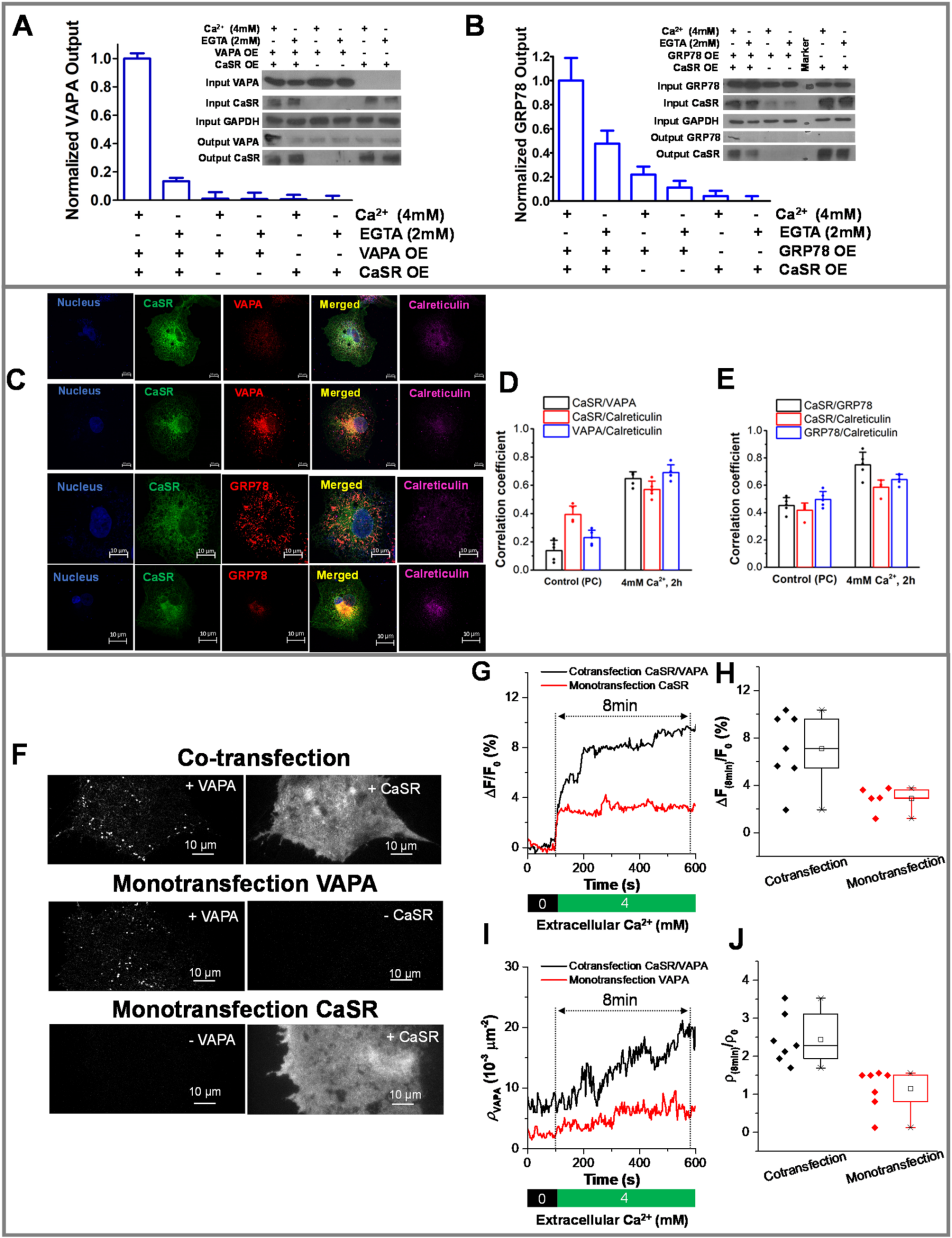
Interactions of CaSR with assistant proteins, VAPA and GRP78, and Ca^2+^_ext_ modulated CaSR-VAPA interdependent cell membrane expression. Co-immunoprecipitation experiments with either Ca^2+^ or EGTA treatments using anti-FLAG antibody on total cell extracts from HEK293 transfected with FLAG-CaSR and VAPA or GRP78 pcDNA3.1 followed by western blot on 50 µg total extract. Analysis with anti-CaSR antibody ((**A,B**) insert; second and fifth panel), anti-VAPA antibody ((**A**) insert, first and fourth panel), anti-GRP78 ((**B**) insert, first and fourth panel) or anti-GAPDH (as control, (**A,B**), third panel) was carried out in triplicate (one representative blot is shown) and is represented in bar graph for output VAPA (**A**) and output GRP78 (**B**). Representative confocal imaging (**C**) showing colocalization of VAPA (red) (in physiological condition (first panel) and 4 mM Ca^2+^ (second panel)), or GRP78 (red) (in physiological condition (third panel) and 4 mM Ca^2+^ (fourth panel)) with CaSR (green) and ER marker (calreticulin, magenta) along the colocalized region (yellow). Scale bar, 2.5 µm. Comparison of pixel intensity correlation of CaSR and ER with VAPA (**D**) or GRP78 (**E**) in confocal images conducted at the regions between cell nuclei and cell membrane boundaries in single cell image sections of Cos7 cells obtained after treatments with physiological Ca^2+^ (DMEM with 2.2 mM) vs 4 mM Ca^2+^. Statistical calculations (Mean ± SD) were determined from five independent cells from different slides obtained with separate transfections (scatter dot). Representative dual-color TIRF images of cells expressing both VAPA and CaSR ((**F**), upper panel), expressing only VAPA ((**F**), middle panel), and expressing only CaSR ((**F**), lower panel). Scale bar, 10 µm. Relative fluorescence intensity changes of membrane expressed CaSR signal in cells with (black line) and without (red line) co-expressing VAPA, represented by a single cell (**G**) and an average representation with n > 5 (**H**). The density of VAPA spots near the cell membrane in cells with (black line) and without (red line) co-expressing CaSR, represented by a single cell (**I**) and an average representation with n > 7 (**J**).

VAPA is a tail anchored VAP family protein localized in the ER, which assists ER in tethering and regulating multiple organelles, such as the Golgi, mitochondria, lysosome, endosome and plasma membrane, by forming contact sites at a close proximity of ~ 30 nm^71^. It has been implicated in Ca^2+^ exchanges^72,73^, including both assistance^36^ or inhibition^37^ of the exit of properly folded proteins through anterograde trafficking from the ER to the Golgi, lipid transfer and in organellar dynamics^71^. Consistent with MS results of 43-fold enrichment of VAPA in the presence of 4 mM Ca^2+^_ex_ (Fig. 2A, Supplemental Table S1), Co-IP and western blot demonstrated Ca^2+^_ex_ dependent enrichment (Fig. 4 A, Supplementary Fig. S8). To establish the interrelationship between Ca^2+^_ex_, CaSR, VAPA and ER, high-resolution confocal microscopy-based pixel intensity correlation analysis was used on images collected on cells treated with differential [Ca^2+^_ex_] (Fig. 4C). Colocalization was 4.3-fold greater under 4 mM Ca^2+^_ex_ treated conditions (Fig. 4C, second panel, Fig. 4D, Supplemental Fig. S4) as compared with the physiological condition (DMEM with 2.2 mM) (Fig. 4C, first panel, Fig. 4D, Supplemental Fig. S4). These colocalizations occur significantly in the ER as illustrated by their concurrence with the ER marker, calreticulin, mostly in the perinuclear region (Fig. 4C, second panel).

### CaSR and VAPA are inter-dependent of each other for the Ca^2+^_ex_ enhanced near-cell membrane expression

To explore the role of VAPA in CaSR expression and function, live cell imaging of Cos7 cells either expressing both EGFP-CaSR and mCherry-VAPA (Fig. 4F, first panel) or only EGFP-CaSR (Fig. 4F, bottom panel) was carried out using total internal reflection fluorescence microscopy (TIRFM). After treatment of 4 mM Ca^2+^_ex_ buffer for 600 s, we observed that the near-cell membrane expression of EGFP-CaSR was enhanced in both co-expressed and mono-expressed cells (Fig. 4G). However, the enhancement was more than twofold higher in co-expressed cells (Fig. 4H). Furthermore, the initial CaSR intensity enhancement rates were similar with or without co-expressing VAPA, but base-to-peak time (t) was longer for co-expressed cells (3.1 ± 1.3 min, n = 7) as compared to mono-expressed cells (1.0 ± 0.6 min, n = 5). We also compared the expression level of mCherry-VAPA near the cell membrane with (Fig. 4F, first panel) or without (Fig. 4F, middle panel) the co-expression of EGFP-CaSR during live cell imaging. More than twofold higher enhancement of near-cell membrane expression of mcherry-VAPA was observed in co-expressed cells (Fig. 4I,J) after applying 4 mM Ca^2+^_ex_ buffer for 600 s. Both the rate and base-to-peak time of the enhancement were larger in co-expressed cells (rate: 1.5 ± 0.5 × 10^–3^ µm^−1^ min^−1^, t = 7.8 ± 0.5 min, n = 7) as compared to that in mono-expressed cells (rate: 0.4 ± 0.3 × 10^–3^ µm^−1^ min^−1^, t = 2.6 ± 1.2 min, n = 7). This not only reiterated that Ca^2+^_ex_ drives CaSR cell membrane expression, but also confirmed that overexpression of VAPA amplified this process. This implied that VAPA could play a prominent role in Ca^2+^_ex_-sensing of CaSR by controlling ER-protein processing and near surface expression.

### Ca^2+^_ex_ controls dynamic interaction of CaSR with protein processing chaperone GRP78 in the ER

We next explored the inter-relationship of Ca^2+^_ex_, CaSR and GRP78 in protein processing as suggested by our novel interactome and gene ontology studies. GRP78 is an Hsp70 family member that act as a major chaperone in protein processing in the ER during various stages, such as the co-translational translocation, calnexin/calreticulin cycle and ER associated degradation (ERAD) (Fig. 3B)^38^. Our MS studies revealed a fivefold Ca^2+^_ex_ dependent enrichment in GRP78 and several complexes related to GRP78 functions such as SEC61, PDIA3, UGGT1, and GRP94^57^ (Fig. 3B, Supplemental Table S1). We further confirmed the enrichment of GRP78 with CaSR in Ca^2+^_ex_ as compared to EGTA-treated conditions by Co-IP and western blot (Fig. 4B, Supplemental Fig. S9). Further, a Ca^2+^_ex_ dependent 1.7-fold increase in the correlation of CaSR with endogenous GRP78 in Cos-7 cells was observed comparing the colocalization between physiological (Figs. 4C, third panel, 4E, Supplemental Fig. S5) and 4 mM Ca^2+^_ex_ treated conditions (Figs. 4C, fourth panel, 4E, Supplemental Fig. S5) using high resolution confocal microscopy-based pixel intensity correlation analysis. These colocalizations occurred significantly in the ER as illustrated by their concurrence with the ER marker, calreticulin (Fig. 4C, fourth panel). Taken together, our studies provided the first verification of Ca^2+^_ex_ dependent interaction of CaSR with the key protein processing chaperone, GRP78, in the ER. We also observed colocalization of GRP78, STUB1, 14-3-3eta and VAPA with CaSR with high Pearson’s coefficients of 0.86, 0.73 and 0.92, respectively, in HEK293 cells (Fig. S6). Further, surface plot analysis of the pixel intensities at colocalized regions demonstrated comparable peaks between CaSR and respective interactors, reaffirming the colocalization (Fig. S6B).

## Discussion

The discovery of CaSR opened the paradigm for a direct role of Ca^2+^_ex_ in signaling, systemic Ca^2+^ homeostasis and consequently, in many calciotropic and non-calciotropic (patho)physiological conditions^10,19^. Numerous studies have reported the role of Ca^2+^_ER_ in protein processing, trafficking, and synthesis. However, both the players and the mechanism regarding how the Ca^2+^_ex_ and the CaSR mediate networks of intracellular Ca^2+^-signaling and related processes remain largely unknown. In our study, we report the first richly annotated CaSR-PPI with 102 interactors, of which 94 are novel. Our comparative proteomics study with low and high Ca^2+^_ex_, along with functional studies and imaging, reveal a Ca^2+^_ex_ dependent alteration of CaSR signaling networks, with two-third of the 102-PPI preferably occurring in high Ca^2+^_ex_. However, the effects of Ca^2+^_ex_ resonates through proteins involved in downstream processes including major Ca^2+^ dependent organellar processes, such as the GPCR signaling, ER related protein processing and trafficking, quality control, endocytosis, and mitochondria transportation (Figs. 2, 3). Notably, this portrays the integrative role of CaSR for crosstalk between Ca^2+^ signaling in the extracellular space and multiple intracellular organelles.

Interestingly, Ca^2+^ binding proteins (CaBP’s) that act as chaperones, buffers, and channels involved in maintaining [Ca^2+^] in intracellular organelles^74^, such as SERCA2b^53^, calnexin (CANX), GRP78, GRP94, PDIA3^75^ and TMCO1^54^, are visualized to be enriched in our proteomics data. Studies have identified a direct link of Ca^2+^ binding SERCA-2b with CaSR^76^ and glucagon receptor (class B, GPCR)^39^. CaSR calcilytic has been shown to attenuate CaSR-induced sarcoplasmic reticulum (SR)-mitochondria crosstalk in a rat cardiomyocyte model^14^. The presence of mitochondrial complexes involved in Ca^2+^_i_ handling and in oxidative stress/early events of apoptosis^53,55,56^, including, VDAC2, SLC25A, TIMM50, ATP5A1, HSPD1 and NDUFA4, and as well as PRDX1in the cytosol, may indicate indirect binding with CaSR, the presence of mitochondrial CaSR^77^, or a role of CaSR in Ca^2+^ mediated oxidative stress^78^. Meanwhile, during trafficking, Ca^2+^_ex_ is known to mobilize the intracellular ER pool of CaSR to the plasma membrane^32,79^. ER chaperones and regulators are dependent on optimal [Ca^2+^_ER_] for proper post-translational processing, folding, and export of proteins^57,80,81^. Our proteomic and gene ontology results delineate a direct link of the Ca^2+^_ex_/CaSR mediated Ca^2+^_ER_ signaling to protein biosynthesis and quality control in the ER based on the enrichment of a significant number of proteins pertaining sequentially to protein-folding, glycosylation and unfolded protein responses in the ER^82^. Identification of interactors such as CANX further validates the biological significance of Ca^2+^_ex_ dependent CaSR-PPI, as CANX is a known CaSR interactor with high Ca^2+^ binding affinity and is known to interact transiently with various soluble non-native conformers of glycoproteins^83^.

Identification of Ca^2+^_ex_ manifested CaSR trafficking associated interactors, such as VAP proteins in the ERGICs is another major implication of our study. VAPA is known to form contact sites between ER and various organelles for Ca^2+^ and lipid regulation^71^ as well as to regulate the anterograde trafficking of properly-folded androgen receptor (a GPCR) and oxysterol-binding protein related protein 3 from the ER to Golgi^37,60,61^. VAPB is an interactor of GPCRs, such as glucagon^39^ and melatonin receptor type 1A^44^. The utility of the CaSR interactome was ascertained by reconfirming the MS data of Ca^2+^_ex_ dependent interactions between GRP78 and VAPA, potentially regulating protein processing in the ER and trafficking, respectively, using orthogonal analysis of Co-IP and western blot. Additionally, the CaSR mediated Ca^2+^_ex_ enhanced VAPA near cell membrane expressions as well as VAPA dependent Ca^2+^_ex_ enhanced CaSR surface expressions were established. This supports the prior knowledge that Ca^2+^_ex_ drives CaSR cell membrane expression. Our work further showed that overexpression of VAPA amplified this process. This implied that VAPA could play a prominent role in Ca^2+^_ex_-sensing of CaSR by controlling ER-protein processing and near surface expression. Similarly, CaSR mediated Ca^2+^ signaling regulated VAPA expression near the cell membrane, hence potentially increasing the ER-plasma membrane contact site establishments. This could explain the agonist driven rapid mobilization of CaSR to cell membranes that has been observed in previous studies^15^.

Our work has independently verified VAPA and GRP78 as CaSR interactors using Co-IP, western blot and imaging analysis. The remaining interactors discussed further in this study remain to be validated with additional direct biochemical studies, such as BRET and FRET. Our result identified p24, which is known to interact with CaSR early in the secretory pathway and assist in transportation to and from the ERGIC^23^, although a single peptide was identified. Additionally, ubiquitously expressed GTPases, Rabs (specifically Rab-1, -7 and -11a) have been previously identified as CaSR interactors^21,84^. Our study complements this list with Rab6^59^. Another protein involved with CaSR trafficking through ERGIC, 14-3-3 ζ, has a distinct function in lowering CaSR membrane expression^27,28,32^. Interestingly, our data shows that 14-3-3 (ζ, θ and ε) binds CaSR at lower [Ca^2+^_ex_] and may retain CaSR to ER in the absence of Ca^2+^ by disruption of the contact. Degradation and endocytosis are major checkpoints for proper functioning of CaSR and the interactome elucidated in this study identifies additional players in these pathways. GPCRs including, adenosine, dopamine-, P2Y, PAR1, glucagon, GABA, mGluRs and CaSR receptor are known to undergo agonist-induced ubiquitination leading to internalization and lysosomal degradation^65^. Ca^2+^ dependence and the role of Ca^2+^ binding protein, such as CaM, in the regulation of ubiquitin is known^85^. Our result showing Ca^2+^_ex_ dependent enrichment of E3 ubiquitin ligases, STUB1 and RanBP2 complements their study. The desensitization of CaSR occurs through endocytosis from the plasma membrane by Ras-related proteins and CLTC. In this study, we report the enrichment of Ca^2+^_ex_ dependent regulators of endocytosis: Rab5^63,64^, Rab18^62^ and CLTC.

CaSR signaling through G-proteins relays Ca^2+^_cyt_ oscillations which code for biological processes. Likewise, we propose that the Ca^2+^_ER_ perturbation dictates protein maturation through the co-translational translocation and calnexin cycle of nascent CaSR polypeptides in the ER. Our data indicate detection of G-proteins, Gα_i_ and Gβ_2_ exclusively in the presence of Ca^2+^_ex_. Heterotrimeric G-proteins are known GPCR interactors^39,44,45^ and Ca^2+^_ex_ activated CaSR transduces diverse downstream signaling through GTP-bound Gα and Gβγ dissociation. Gα_i_ mediates inhibition of the cAMP dependent pathway through inhibition of adenylate cyclase^86^. On the other hand, Gβ_2_ may modulate ion channels^87^, anterograde trafficking^15^, microtubule assembly^88^ and ubiquitination of GPCR^89^. Upregulation of the signal transducing G-proteins reaffirms the direct correlation of Ca^2+^_ex_ and CaSR downstream signaling.

Our studies using MS coupled with IP also have some limitations in detecting transient interactions. Recruitment of some known CaSR interactors were missing in our report. Some of these ‘interactions’ are unlikely to be direct interactions, but could be part of a signaling complex, for example with clathrin. There are possible flaws of IP: that it is unlikely to capture transient and low affinity binding; that it does not show direct interactions; and that use of whole cell lysates can yield false positives as two proteins that are never in proximity can serendipitously interact within lysates. Our Co-IP results obtained with exposure of cells with 4 mM Ca^2+^ for 2 h Ca^2+^ treatment may possibly depict physiological interactions in tissues maintained at higher [Ca^2+^], such as bone environment (reported as high as 10 mM)^90^. Our interactome may also depict the severe physiological implication of high Ca^2+^ conditions, such as during severe hypercalcemia where the serum calcium levels are above 2.65 mM^91^. In our study we have aimed to capture proteins that interact with CaSR throughout its life cycle from signaling, internalization, endocytosis, synthesis to insertion through agonist-derived insertional signaling (ADIS)^15^. Our previous study reported that two-thirds of the CaSR remains on the cell surface and only 30% is internalized with 4 mM Ca^2+^ exposure for 2 h^35^. In order to understand the physiological relevance, we: (i) used live cell imaging (Fig. 4F–J) spanning 10 min of Ca^2+^ treatment and validated the significance of interaction of CaSR with important interactors such as the VAPA and captured the effect of Ca^2+^ on CaSR signaling; (ii) captured the temporal effect of CaSR activation in ER-Ca^2+^ change within few seconds (Fig. 3C); and (iii) compared the positive correlation coefficients of CaSR-VAPA (Fig. 4D) and CaSR-GRP78 (Fig. 4E) in physiological conditions to that with 2 h Ca^2+^ treated conditions. Recruitment of some known CaSR interactors could have been missed in our report due to interactions that may have occurred during the earlier CaSR activation, such as those with G-proteins, AP2 and beta-arrestins. This lack of detection could be reflective of poor abundance in cells for instance, as observed for G-proteins including Gq/11, Gi_1_, Gi_3_, Gs, Gγ_12_ and Gγ_5_, which all had the average number of peptides counts less than 1 in the whole cell lysate MS/MS results. On the other hand, Gi_2_, Gβ_2_, and Gβ_2_-like-1 proteins had higher average peptide counts from 3 to 27, resulting in robust detection. Further, filamin A and CaM were detected with robust peptide counts but with no significant changes between the positive and negative controls, implying un-specific enrichment. CaM, filamin-A, and Gβγ, are known to bind to the same binding region within the mGluR_7a_ C terminus^92^. This could be one of the reasons why our analysis only detected Gβγ-subunits. Also, the observed examples of enrichment could have been affected by binding affinities and transient interactions, which cannot be captured without proximity labeling. Further, we cannot negate the fact that the putative CaSR binding partners detected may be due to direct or indirect interactions. Proteins such as clathrin could be part of a signaling complex. Therefore, our results may not be a complete inventory of the putative CaSR interactome. Another aspect to note is that we achieved the interactome in HEK293 cells that were overexpressed with CaSR-FLAG. The interactions may differ depending on cell types and in endogenously CaSR-expressed cells in physiological conditions. Thus, we performed additional colocalization studies in Cos-7 cells expressing endogenous VAPA and GRP78 (Fig. 4C–E). The result showed a positive correlation with CaSR and GRP78, and CaSR and VAPA in physiological condition which increases by two and fourfold after Ca^2+^ treatment. Figure S6 presents evidence regarding the correlation as we demonstrate high colocalizations between CaSR and endogenous proteins such as GPR78, CHIP, 14-3-3 and VAPA in a different cell line, i.e., HEK293. It is anticipated that additional live cell imaging and Co-IP studies will further support these results.

However, our work provides a powerful resource for elucidating Ca^2+^_ex_/CaSR signaling and identifying putative targets for CaSR-based therapeutics. It provides a platform for studies in many directions to understand Ca^2+^_ex_ dependent mechanisms for putative interactors. In this study we have been able to provide the first extensive PPI network for CaSR and demonstrate the association of Ca^2+^_ER_ release as a response to Ca^2+^_ex_ via CaSR. Our work surmises the potential roles of Ca^2+^_ex_ in CaSR interactomes related to (i) signal transduction, (ii) maturation of nascent polypeptides, (iii) trafficking, (iv) quality control through degradation, (v) desensitization and (vi) Ca^2+^_i_ handling. This study expands the repertoire of the CaSR interactome through the identification of 94 putative novel CaSR interactors. We were also able to recapitulate eight previously identified interactors of CaSR and several interactors previously known for other members of GPCR family, indicating overlapping signaling cascade and intracellular processes. Additionally, our study provides further evidence of Ca^2+^_ex_ dependent association of CaSR with important trafficking and protein processing proteins, VAPA and GRP78. Taken together, our work provides a powerful resource for research in Ca^2+^_ex_/CaSR signaling and putative targets for CaSR-based therapeutics.

## Materials and methods

### Plasmids and reagents

Empty pcDNA3.1, human CaSR with FLAG-tag (FLAG-hCaSR) (between Asp^371^ and Thr^372^) in pcDNA3.1 and EGFP-CaSR (provided by Dr. Chen Zhang, La Jolla Institute of Allergy and Immunology, CA) were used for transfection for negative and positive controls, respectively. pEGFPC1-hVAPA and pcDNA3.1 GRP78 were obtained from Addgene. mCherry-VAPA was constructed from pEGFPC1-hVAPA (Addgen) and mCherry in pcDNA3.1. The EGFP was removed from pEGFPC1-hVAPA between restriction sites Nhel (895) and Xhol (985), and mCherry with the paired sticky ends was fused to the N terminus of VAPA. For TIRF imaging, mCherry-VAPA and EGFP-CaSR were used for the transfection. The purified plasmids were prepared using a Mini Prep Kit (Qiagen, Toronto, Canada).

### Cell culture

Monolayer culture of HEK293 cells and Cos-7 cells were purchased from American Type Culture Collection (ATCC CRL-1573) and cultured with high glucose (4.5 g/L) Dulbecco’s Modified Eagle Medium (DMEM) (Invitrogen, Carlsbad, California) supplemented with 10% fetal bovine serum (FBS, Atlanta Biologicals) in a humidified environment at 37 °C with 5% CO_2_. For MS, transient transfection with 6 µg of empty pcDNA3.1 or FLAG-hCaSR was performed in 100 mm dishes using lipofectamine 3000 in the same medium following the manufacturer’s protocol (Invitrogen, Carlsbad, California). At 48 h post-transfection, cells were washed with Hank’s Balanced Salt Solution (HBSS) (Sigma-Aldrich, Canada) at 37 °C, incubated in starving medium low glucose DMEM, 0 mM Ca^2+^ with 0.1% BSA for 30 min and finally treated with various [Ca^2+^] or 2 mM EGTA at various time points. For TIRF imaging, Cos-7 cells were cultured on 22 mm × 40 mm coverslips pre-coated with Poly-l-lysine solution. Transient transfection with 1 µg of mCherry-VAPA or EGFP-CaSR was performed for the mono-transfection, and 1 µg of mCherry-VAPA plus 1 µg of EGFP-CaSR was performed for the co-transfection in each coverslip using lipofectamine 3000 in the same medium following the manufacturer’s protocol (Invitrogen, Carlsbad, California). Cell images were collected at 48 h post-transfection.

### Antibodies

Anti-FLAG M2, mouse (F1804, Sigma-Aldrich, Canada) was used to precipitate the CaSR/interactor complex. Anti-CaSR C0493, mouse (Abcam, Cambridge, MA, USA) and Anti-GAPDH mouse (Abcam, Cambridge, MA, USA) were used for western blot. Anti-VAPA (15275-1-AP, rabbit (ProteinTech, Illinois, USA) and anti-GRP78 (ab21685), rabbit (Abcam, Cambridge, MA, USA) were used for western blot and immunostaining. Goat anti-rabbit IgG-AP conjugate (1706518, BioRad) and goat anti-mouse IgG-AP conjugate (1706520, BioRad) were used as secondary antibodies for western blot. Anti-GFP antibody mouse (ab13970, Abcam) was used to immunostain GFP-CaSR. Donkey anti mouse IgG (H + L) Alexa fluor 647 (A31571, Invitrogen), goat anti-mouse IgG (H + L) Alexa Fluor 488 (A32723, Invitrogen), goat anti-mouse IgG (H + L) Alexa Fluor 555 (A-21422, Thermo Fisher Scientific) and goat anti-chicken IgY H&L Alexa Fluor 488 (ab150173, Abcam) were used as secondary antibodies for immunostaining.

### Total protein extracts

Transfected HEK293 cells from 90 to 100% confluent 100 mm dishes were harvested after the treatment with 4 mM Ca^2+^ or 2 mM EGTA for 2 h. These were then washed three times with ice cold phosphate-buffered saline (PBS) with 0 mM Ca^2+^. Next, 600 μL of 10 mM sodium β-glycerophosphate, 50 mM Tris–Cl (pH 7.4), 150 mM NaCl, 1% Triton X-100, 2 mM Na_3_VO_4_, 50 mM NaF, 10 mM sodium pyrophosphate supplied with proteinase inhibitor cocktail (Roche, Basel, Switzerland) was used to lyse cells for 30 min in ice with frequent vortex, followed by centrifugation to pellet cell debris. Cleared cell lysates were subjected to anti-FLAG immunoprecipitation prior to immunoblotting.

### Coimmunoprecipitation (Co-IP)

For each condition, a total of 1.0 mg of total protein was used as measured by Bradford assay. Anti-FLAG antibody (10ug) and Protein G Dynabeads (10003D, Thermo Fisher) were incubated for 30 min at room temperature in 200 µL PBS and 0.01% Tween 20, and washed once with lysis buffer. Magnetic beads were used as they give a low background of the contaminant proteins^93^. Antigen was added and incubated for 10 h at 4 °C. The next day, beads were washed two times with lysis buffer and two times with PBS. The 10% beads were then suspended in 30 µL of 2× sample buffer with 5% β-mercaptoethanol and heated for 10 min at 100 °C. The rest of the 90% bead was used for on bead digestion and LC–MS/MS. For VAPA and GRP78 validations, 100% beads were used for western blot.

### Immunoblotting

A total input protein of 50 µg and 30 µL of the 10% bead samples were loaded in 8.5% acrylamide gels and subjected to sodium dodecyl sulfate–polyacrylamide gel electrophoresis (SDS-PAGE) to separate proteins, then transferred to nitrocellulose membranes. The membranes were blocked with 3% nonfat milk (w/v) in TBS for 2 h at room-temperature, with constant shaking. The antibodies of interest were diluted in 3% non-fat milk (w/v) and 0.2% Tween-20 in TBS (TBST). Anti-CaSR C0493, mouse was used at 1:700 dilution and HRP-conjugated mouse secondary antibody was used (Sigma-Aldrich, United States) at 1:1500 dilution to probe CaSR. For GAPDH, anti-GAPDH mouse was used at 1:2000 dilution and HRP-conjugated mouse secondary antibody was used (Sigma-Aldrich, United States) at 1:3000 dilution. For VAPA, anti-VAPA rabbit was used at 1:500 dilution, and HRP-conjugated rabbit secondary antibody was used (Sigma-Aldrich, United States) at 1:1500 dilution. For GRP78, anti-GRP78 rabbit was used at 1:1000 dilution, and HRP-conjugated rabbit secondary antibody was used (Sigma-Aldrich, United States) at 1:1500 dilution Membranes were incubated with the primary and secondary antibodies for 1 h at room-temperature with constant shaking, and finally washed with TBST. Secondary antibody was visualized using ECL detection reagents (GE healthcare, Little Chalfont, UK) developed on X-OMAT™ imaging film (Kodak, Rochester, NY).

### On-bead digestion on Co-IP samples

On-bead digestion was carried out at room temperature according to the published protocol^94^. The IP beads were washed three times with 1× PBS to remove detergents. To the bead, digestion buffer (50 mM NH_4_HCO_3_) was added, and the mixture was then treated with 1 mM dithiothreitol (DTT) for 30 min, followed by 5 mM iodoacetimide (IAA) for 30 min in the dark. Proteins were digested overnight with 0.5 µg of lysyl endopeptidase (Wako) and were further digested overnight with 1 µg trypsin (Promega). Resulting peptides were desalted with HLB column (Waters) and were dried under vacuum.

### LC–MS/MS on Co-IP samples

Peptides were analyzed with Nano-High Pressure Liquid Chromatography-Tandem Mass Spectrometry (nano-LC–MS/MS). Briefly, the peptides were loaded onto an in-house packed column (40 cm long × 75 μm ID × 360 OD, Dr. Maisch GmbH ReproSil-Pur 120 C18-AQ 3.0 µm beads) analytical column (Thermo Scientific) using a Dionex nanoLC system (Thermo Scientific). The column output was connected to a Q Exactive Plus mass spectrometer (Thermo Scientific) through a nanoelectrospray ion source. The mass spectrometer was controlled by Xcalibur software (Thermo, 4.0.27.19) and operated in the data-dependent mode in which the initial MS scan recorded the mass-to-charge ratios (m/z) of ions over the range of 350–1750 at a resolution of 70,000 with a target value of 1 × 10^6^ ions and a maximum injection time of 100 ms. The 10 most abundant ions were automatically selected for subsequent higher-energy collision dissociation (HCD) with the energy set at 28 NCE. The MS/MS settings included a resolution of 35,000, a target value of 5 × 10^5^ ions, a maximum integration time of 108 ms, and an isolation window was set at 3.0 m/z. Ions with undetermined charge, z = 1, 8, and z > 8 were excluded.

### LC–MS/MS on total cell lysate

Each sample was analyzed by nano LC–MS/MS with a Waters NanoAcquity HPLC system interfaced to a ThermoFisher Q Exactive. Peptides were loaded on a trapping column and eluted over a 75 μm analytical column at 350 nL/min; both columns were packed with Luna C18 resin (Phenomenex). The mass spectrometer was operated in the data-dependent mode in which the initial MS scan recorded the mass-to-charge ratios (m/z) of ions over the range of 300–1600 at a resolution of 70,000 with a target value of 3 × 10^6^ ions and a maximum injection time of 120 ms. The 15 most abundant ions were automatically selected for subsequent higher-energy collision dissociation (HCD) with the energy set at 25 NCE. The MS/MS settings included a resolution of 17,500, a target value of 1 × 10^5^ ions, a maximum integration time of 120 ms, and an isolation window set at 1.5 m/z. Ions with undetermined charge, z = 1, and z > 8 were excluded.

### Protein identification, quantitation and statistical analysis

LC–MS/MS Q-Extractive Orbitrap was used. Raw data files were analyzed with MaxQuant version 1.6.3.3 (Thermo Foundation 2.0 for RAW file reading capability) using an established Maxquant setup^95^. Intensities of each protein for each treatment condition were averaged from three negative control IP samples and three CaSR IP samples. The missing values were imputed as previously described using Perseus^96^. The following stringencies were employed to ensure robust upregulation, reproducibility, and detection**:** (i) treatments and IP for the positive and negative controls were performed in triplicates; (ii) identified proteins were at least two-fold enriched in the CaSR transfected samples over the negative controls, i.e., log_2_ (HEK293 + CaSR-FLAG pcDNA/Hek293 + pcDNA3.1) ≥ 1.00; (iii) P-values were calculated using a two-sided Student’s t-test, with a null hypothesis that there was no difference in protein relative abundance between the two groups, and a two tailed t-test with P ≤ 0.05 between the groups being statistically significant; (iv) proteins had a minimum peptide spectrum match (PSM) of two for at least two replicates, and; (v) proteins were identified with at least one unique peptide.

### Functional annotation of identified protein partners

The Database for Annotation, Visualization, and Integrated Discovery (DAVID)^46–48^, version 6.8, was used for the functional annotation and analysis of enrichment of 102 proteins identified as putative CaSR interactors. The set of total proteins identified and quantified (n = 623) was used as the background. The 102 proteins of interest and background lists were compared in each functional cluster. A two-tailed modified fisher’s exact test (EASE score of 1) with classification stringency at “medium” was employed to generate statistically significant enrichment annotations and to categorize them under annotation terms: cellular compartments, biological processes, molecular function, and KEGG pathways. Correction for multiple hypothesis testing was carried out using standard false discovery rate control methods. A Bonferroni-corrected P ≥ 0.05 and an enrichment ≥ 1.3 were used as cut-offs^47,48^. Similar analysis was performed on 102 putative CaSR interactors and 11 literature-curated CaSR interactors to represent a comprehensive CaSR PPI. Groups with annotations comprising of cellular compartments, biological processes and molecular function were presented with Cytoscape 3.7.0^97^.

Visual representations of the PPI network for putative 102 CaSR interactors (enriched in both Ca^2+^ and EGTA conditions) and 11 literature-curated were generated using Search Tool for the Retrieval of Interacting Genes/Proteins (STRING) version 10.0^52^. Interactions were identified and visualized among the 102 putative CaSR interactors (Homo sapiens). STRING used evidence from experimental and knowledge-based databases to provide confidence in functional associations or interaction through the Edge Confidence. Size of colored nodes represent evidence of known or predicted 3-dimensional protein structures.

### Immunostaining

HEK293 cells and Cos-7 cells were grown on 20 × 20 mm coverslips placed in 6-well plates, then transfected with 1.2 µg FLAG-hCaSR and EGFP-CaSR DNA, respectively, and allowed to grow for 48 h prior to immunostaining. Cells were washed with ice cold PBS and fixed with 3.7% formaldehyde for 15 min at room temperature, followed by wash with PBS three times. Cells were permeabilized using 0.2% Triton X in PBS for 10 min at room temperature. HEK293 cells were incubated with mouse anti-FLAG monoclonal antibody at 1:1000 dilution and goat anti-mouse IgG (H + L) Alexa Fluor 488 secondary antibody (A32723, Invitrogen) for 1 h each at room temperature to stain FLAG-CaSR. Cos7 cells were incubated with anti-GFP antibody at 1:2000, anti-calreticulin antibody at 1:200, and anti-VAPA antibody at 1:125 or anti-GRP78 antibody at 1:100 in PBS with 3% BSA at room temperature for 1 h. The cos-7 cells were subsequently washed with PBS and stained with secondary antibodies goat anti-chicken IgY H&L Alexa Fluor 488 (ab150173, Abcam), donkey anti mouse IgG (H + L) Alexa fluor 647 (A31571, Invitrogen) and goat anti-mouse IgG (H + L) Alexa Fluor 555 (A-21422, Thermo Fisher Scientific), respectively for 1 h at room temperature. Nuclei were stained with 4′,6-diamidino-2-phenylindole.

### Live cell imaging using total internal reflection fluorescence microscopy (TIRFM)

Fluorescence images were collected using a Nikon Ti-E invert microscope equipped with Nikon 100× TIRF objective and Andor IXon Ultra 888 EMCCD camera. The cells were imaged under total internal reflection fluorescence microscopy with an imaging speed of 1 frame per second. The excitation wavelengths for EGFP-CaSR and mCherry-VAPA were 488 and 561 nm, and a Quad Band filter set (TRF89901v2, Chroma) was used for filtering out the fluorescence background. A home-built dual-color imaging device with a long pass dichroitic mirror at 561 nm (Semrock) was used to split the fluorescence signals from EGFP-CaSR and mCherry-VAPA, which allowed for simultaneous collection of fluorescence images from both proteins. Another pair of fluorescent filters (515/30, Semrock and 620/60, Chroma) were also used for dual-color imaging to minimize the color cross talk. For live cell imaging, Cos-7 cells on the coverslips were washed three times with 0 mM Ca^2+^ buffer, then mounted in flow chamber with 0 mM Ca^2+^ buffer as the initial condition. Next, 4 mM Ca^2+^ buffer was added at 100 s, and then live cell images were collected for 600 s.

### Epifluorescence imaging of CaSR mediated ER Ca^2+^ dynamics using G-CatchER^+^ and Fura-red

HEK293 cells transfected with G-CatchER^+^ and wt-CaSR were incubated with Fura-red for 30 min at 37 °C then washed with 2 mL of physiological Ringer buffer (10 mM HEPES, 140 mM NaCl, 5 mM KCl, 1.2 mM MgCl_2_, 1.8 mM CaCl_2_ at pH 7.4). The coverslips were mounted on a bath chamber and placed on the stage of a Leica DM6100B inverted microscope with a Hamamatsu cooled EM-CCD camera and illuminated with a Till Polychrome V Xenon lamp. Cells were illuminated at 488 nm and 550 nm, in real-time, as cells were exposed to 0.5 mM Ca^2+^for 200 s, followed by 4 mM Ca^2+^ for another 800 s and back to 0.5 mM Ca^2+^ for additional 200 s.

### Image analysis

Multicolor fluorescence images of nucleus (Ex: 350 nm; Em: 470 nm), CaSR (Ex: 490 nm; Em: 525 nm), VAPA/GRP78 (Ex: 555 nm; Em: 565 nm), and ER (Ex: 650 nm; Em: 665 nm) were taken using a Zeiss LSM780 confocal microscope. To analyze the colocalization between each imaging channels, a self-written MATLAB script was used. Briefly, regions of interest (ROIs) were first identified using merged images from all imaging channels. Next, Pearson coefficients were calculated between pixel intensities at the same ROI in green, red, and purple channels. The same image analysis method was used over different cell treatment conditions.

## Supplementary Information

Supplementary Information 1.

Supplementary Information 2.

Supplementary Information 3.

## Data Availability

All data needed to evaluate the conclusions in the paper are present in the paper and/or the Supplementary Materials. The raw mass spectrometry data will be deposited to a public repository prior to publication.
