# Supplementary material for: Extracellular calcium alters calcium-sensing receptor network integrating intracellular calcium-signaling and related key pathway
Source: Sci Rep. 2021 Oct 18;11:20576. doi: 10.1038/s41598-021-00067-2 (PMC8523568; doi:10.1038/s41598-021-00067-2)
Supplement: Supplementary file 1 — Supplementary Information 1. [file 41598_2021_67_MOESM1_ESM.pdf]

Supplemental Table 1. Heat Map of 106 putative CaSR interactors

Protein names	Gene names	Log_2 [positive/negative control] - Ca^{2+}	Log_2 [positive/negative control] -EGTA	Log_2 (Ca^{2+}/EGTA)-Positive control
Vesicle-associated membrane protein-associated protein A	VAPA	5.19	-0.53	5.42
UDP-glucose:glycoprotein glucosyltransferase 1	UGGT1	4.79	0.20	4.48
Guanine nucleotide-binding protein G(I)/G(S)/G(T) subunit beta-2	GNB2	5.02	1.00	4.43
E3 SUMO-protein ligase RanBP2	RANBP2	3.86	0.39	4.39
Tyrosine-protein phosphatase non-receptor type;Tyrosine-protein phosphatase non-receptor type 1	PTPN1	3.03	-2.00	4.24
Leucine-rich repeat-containing protein 59	LRRC59	6.14	2.80	4.16
Signal recognition particle receptor subunit alpha	SRPR	3.29	-0.41	4.11
Ras-related protein Rab-5C	RAB5C	4.31	1.18	4.10
Nuclear pore complex protein Nup155	NUP155	4.71	0.36	4.00
Signal recognition particle receptor subunit beta	SRPRB	6.69	2.84	3.74
Guanine nucleotide-binding protein subunits G(k); G(i) alpha-1; G(i) alpha-2; G(o) alpha	GNAI3	6.75	1.04	3.64
Membrane-associated progesterone receptor component 1	PGRMC1	2.95	0.59	3.02
Ancient ubiquitous protein 1	AUP1	3.67	1.27	2.87
ATP synthase protein 8	MT-ATP8	2.66	1.16	2.73
Erlin-1	ERLIN1	2.84	0.22	2.70
FAS-associated factor 2	FAF2	4.61	1.89	2.48
Vesicle-associated membrane protein-associated protein B/C	VAPB	3.34	1.00	2.37
Erlin-2	ERLIN2	7.38	4.43	2.35
78 kDa glucose-regulated protein	HSPA5	4.25	2.69	2.30
Sodium/potassium-transporting ATPase subunit alpha-1; alpha-3; alpha-2	ATP1A1	4.76	1.81	2.20
S-phase kinase-associated protein 1	SKP1	1.56	0.46	2.00
Ras-related protein Rab-6A;Ras-related protein Rab-6B;Ras-related protein Rab-39A	RAB6B	3.19	1.26	1.96
Protein disulfide-isomerase A3	PDIA3	5.19	3.28	1.60
Urotensin-2	UTS2	4.46	3.10	1.58
GTP-binding nuclear protein Ran	RAN	3.07	2.23	1.52
Lamina-associated polypeptide 2, isoforms beta/gamma;Thymopoietin;Thymopentin;Lamina-associated polypeptide 2, isoform alpha;Thymopoietin;Thymopentin	TMPO	3.17	3.40	1.51
Ras-related protein Rab-18	RAB18	2.15	1.70	1.51
Peptidyl-prolyl cis-trans isomerase FKBP8;Peptidyl-prolyl cis-trans isomerase	FKBP8	4.32	3.22	1.50
Very-long-chain enoyl-CoA reductase	TECR	3.63	2.51	1.49
Eukaryotic initiation factor 4A-I;Eukaryotic initiation factor 4A-II;Eukaryotic initiation factor 4A-II, N-terminally processed	EIF4A1	2.26	1.80	1.49
ATP synthase subunit alpha, mitochondrial	ATP5A1	3.12	3.58	1.41
Dolichyl-diphosphooligosaccharide--protein glycosyltransferase subunit 1	RPN1	7.07	5.51	1.35
Protein transport protein Sec61 subunit beta	SEC61B	3.40	2.18	1.33
WD repeat-containing protein 6	WDR6	3.53	3.46	1.30
Hemoglobin subunit beta;LVV-hemorphin-7;Spinorphin	HBB	2.26	-0.37	1.22
Sarcoplasmic/endoplasmic reticulum calcium ATPase 2	ATP2A2	6.81	6.36	1.18
Transducin beta-like protein 2	TBL2	3.73	3.29	1.15
Endoplasmin	HSP90B1	4.18	2.93	1.10
60 kDa heat shock protein, mitochondrial	HSPD1	3.27	3.03	1.09
Clathrin heavy chain;Clathrin heavy chain 1	CLTC	2.37	1.92	1.05
Dolichyl-diphosphooligosaccharide--protein glycosyltransferase 48 kDa subunit	DDOST	2.21	1.93	1.02

Complement component 1 Q subcomponent-binding protein, mitochondrial	C1QBP	0.66	1.70	0.98
Elongation factor 1-gamma	EEF1G	3.56	3.69	0.95
Elongation factor 2	EEF2	3.03	2.98	0.95
40S ribosomal protein S5;40S ribosomal protein S5, N-terminally processed	RPS5	2.22	3.08	0.90
40S ribosomal protein S27	RPS27	3.49	3.93	0.90
Elongation factor 1-alpha 1;Putative elongation factor 1-alpha-like 3;Elongation factor 1-alpha	EEF1A1	1.95	1.39	0.90
Trifunctional enzyme subunit beta, mitochondrial;3-ketoacyl-CoA thiolase	HADHB	1.16	1.79	0.89
DnaJ homolog subfamily A member 1	DNAJA1	3.16	4.37	0.86
40S ribosomal protein S27;40S ribosomal protein S27-like	RPS27L	3.73	2.87	0.82
Microsomal glutathione S-transferase 2	MGST2	3.74	3.70	0.81
Lamin-B receptor	LBR	3.48	3.21	0.81
Importin subunit beta-1	KPNB1	2.08	3.94	0.79
E3 ubiquitin-protein ligase CHIP	STUB1	3.06	2.43	0.78
Neutral alpha-glucosidase AB	GANAB	7.22	6.63	0.76
Very-long-chain (3R)-3-hydroxyacyl-CoA dehydratase 3	HACD3	4.42	4.97	0.72
Calnexin	CANX	6.57	6.69	0.71
Peroxiredoxin-1	PRDX1	0.89	1.30	0.71
Heat shock protein HSP 90-alpha	HSP90AA1	3.24	2.84	0.69
Up-regulated during skeletal muscle growth protein 5	USMG5	3.09	1.57	0.68
Dolichyl-diphosphooligosaccharide--protein glycosyltransferase subunit 2	RPN2	4.68	4.79	0.68
Monocarboxylate transporter 1	SLC16A1	4.95	4.48	0.66
Cytochrome c oxidase subunit NDUFA4	NDUFA4	3.22	4.54	0.66
Voltage-dependent anion-selective channel protein 2	VDAC2	3.11	3.03	0.66
Proteasome subunit alpha type;Proteasome subunit alpha type-4;Proteasome subunit beta type	PSMA4	2.24	2.53	0.63
ADP/ATP translocase 2;ADP/ATP translocase 2, N-terminally processed	SLC25A5	2.11	2.35	0.60
Transmembrane and coiled-coil domain-containing protein 1	TMCO1	4.49	5.66	0.58
40S ribosomal protein S14	RPS14	0.81	2.16	0.58
Phosphate carrier protein, mitochondrial	SLC25A3	3.63	4.38	0.52
ADP/ATP translocase 3;ADP/ATP translocase 3, N-terminally processed	SLC25A6	2.03	2.65	0.51
40S ribosomal protein S20	RPS20	0.75	1.18	0.47
Poly(rC)-binding protein 1	PCBP1	1.99	2.14	0.44
Heat shock cognate 71 kDa protein	HSPA8	2.96	3.57	0.43
DnaJ homolog subfamily C member 7	DNAJC7	7.08	8.23	0.38
Extracellular calcium-sensing receptor	CASR	11.61	14.32	0.30
40S ribosomal protein S3	RPS3	1.06	1.43	0.29
ATP synthase subunit gamma, mitochondrial	ATP5C1	1.07	1.92	0.28
Heat shock protein HSP 90-beta	HSP90AB1	2.34	3.81	0.26
Heat shock 70 kDa protein 1A	HSPA1A	2.03	2.88	0.26
40S ribosomal protein S16	RPS16	1.28	0.93	0.26
60S ribosomal protein L22	RPL22	0.63	1.11	0.24
BAG family molecular chaperone regulator 2	BAG2	6.08	5.83	0.17
DNA-dependent protein kinase catalytic subunit	PRKDC	1.53	4.98	0.13
Mitochondrial import inner membrane translocase subunit TIM50	TIMM50	3.23	2.37	0.13
Glyceraldehyde-3-phosphate dehydrogenase	GAPDH	2.23	2.38	0.12
60S ribosomal protein L38	RPL38	1.07	1.05	0.11
60S ribosomal protein L23	RPL23	1.21	1.69	0.09
Tubulin beta-4B chain;Tubulin beta-4A chain	TUBB4B	1.64	3.57	0.09
RalA-binding protein 1	RALBP1	7.16	-0.21	0.08
GPALPP motifs-containing protein 1	GPALPP1	9.40	9.03	0.07
Tubulin beta chain;Tubulin beta-2B chain;Tubulin beta-2A chain	TUBB	1.92	3.48	0.01
Tubulin beta-8 chain	TUBB8	2.61	4.73	-0.02

14-3-3 protein gamma;14-3-3 protein gamma, N-terminally processed	YWHAG	2.04	2.75	-0.04
Tubulin alpha-1B chain;Tubulin alpha-4A chain	TUBA1B	1.85	3.28	-0.12
ATP synthase subunit f, mitochondrial	ATP5J2	3.08	4.90	-0.18
Tricarboxylate transport protein, mitochondrial	SLC25A1	2.51	5.30	-0.33
40S ribosomal protein S17-like;40S ribosomal protein S17	RPS17	0.03	4.97	-0.53
Activated RNA polymerase II transcriptional coactivator p15	SUB1	1.53	3.13	-0.53
14-3-3 protein epsilon	YWHAE	4.42	4.91	-0.80
Tubulin alpha-1C chain	TUBA1C	3.16	4.54	-0.99
14-3-3 protein theta	YWHAQ	4.68	7.46	-1.08
14-3-3 protein zeta/delta	YWHAZ	3.72	4.54	-1.38
THO complex subunit 4	ALYREF	-0.94	1.49	-1.69
