# Supplementary material for: Extracellular calcium alters calcium-sensing receptor network integrating intracellular calcium-signaling and related key pathway
Source: Sci Rep. 2021 Oct 18;11:20576. doi: 10.1038/s41598-021-00067-2 (PMC8523568; doi:10.1038/s41598-021-00067-2)
Supplement: Supplementary file 2 — Supplementary Information 2. [file 41598_2021_67_MOESM2_ESM.pdf]

Supplemental Table 3. 623 identified proteins by MS during Co-IP against CaSR, including Ca^{2+} and EGTA treatments

Majority protein IDs	Protein names	Gene names	Number of	Peptides	Razor + unique	Unique	Sequence	Mol.	Q-	Score
F8VZ44;Q9NRG9-2;Q9NRG9;F8VUB6;H3BU82	Aladin	AAAS	5	2	2	2	4.5	45.634	0.008741	1.9958
P28288-2;P28288;P28288-3	ATP-binding cassette sub-family D member 3	ABCD3	3	2	2	2	4.7	62.725	0	3.312
J3QSX6;H0Y3K7;F8W8M4;A0A0A0MRL6;O14639- 4;O14639-3;O14639-5;O14639-2;O14639-	Actin-binding LIM protein 1	ABLIM1	12	2	2	2	7.2	46.07	0	4.512
B7Z9I1;Q5T4U5;P11310;P11310-2	Medium-chain specific acyl-CoA dehydrogenase,	ACADM	4	2	2	2	6.5	42.426	0.008078	1.612
S4R3H4;E7EQT4;Q9UKV3;Q9UKV3- 5;G3V3B0;Q9UKV3-3;Q9UKV3-2	Apoptotic chromatin condensation inducer in the nucleus	ACIN1	9	24	24	24	35.7	145.44	0	323.31
O95573;H7C260	Long-chain-fatty-acid--CoA ligase 3	ACSL3	2	2	2	2	4.2	80.419	0.008547	1.8129
P60709	Actin, cytoplasmic 1;Actin, cytoplasmic 1, N-terminally	ACTB	14	26	1	1	89.1	41.736	0	255.3
P68032;P68133;P62736;A6NL76;P63267	Actin, alpha cardiac muscle 1;Actin, alpha skeletal muscle;Actin, aortic smooth muscle;Actin, gamma-enteric	ACTC1;ACTA1;ACTA2; ACTG2	9	17	5	5	53.3	42.019	0	130.09
P63261;I3L3I0;I3L1U9;I3L4N8	Actin, cytoplasmic 2;Actin, cytoplasmic 2, N-terminally	ACTG1	7	26	26	1	89.1	41.792	0	323.31
O43707;O43707-2	Alpha-actinin-4	ACTN4	20	5	5	5	7.5	104.85	0	6.4945
P61160;F5H6T1;P61160-2	Actin-related protein 2	ACTR2	3	4	4	4	12.2	44.76	0	7.3054
P61158;B4DXW1	Actin-related protein 3	ACTR3	12	6	6	6	26.3	47.371	0	38.883
Q8N142;Q8N142-2	Adenylosuccinate synthetase isozyme 1	ADSSL1	2	1	1	1	3.9	50.208	0.008591	1.8349
J3QRD1;P51648;P51648-2;J3QS00;I3L1M4	Fatty aldehyde dehydrogenase	ALDH3A2	5	2	2	2	6.4	44.648	0.008183	1.6712
Q86V81;E9PB61	THO complex subunit 4	ALYREF	2	6	6	6	36.2	26.888	0	58.093
H3BRK9;R4GNG2;Q9UKV5	E3 ubiquitin-protein ligase AMFR	AMFR	3	1	1	1	10.4	32.768	0.007092	2.0932
O95782-2;O95782	AP-2 complex subunit alpha-1	AP2A1	2	1	1	1	2.8	105.36	0	2.3669
A0A087WYD1;A0A087WU93;A0A087WZQ6;A0A0 87X253;P63010-3;P63010;P63010-2	AP-2 complex subunit beta	AP2B1	7	1	1	1	4.1	70.916	0	2.3364
A0A087WTM7;P04114	Apolipoprotein B-100;Apolipoprotein B-48	APOB	2	2	2	2	0.9	489.83	0	2.5251
C9JPM4;C9JAK5;P18085;F5H0C7	ADP-ribosylation factor 4	ARF4;ARF3	4	2	2	1	23.6	14.553	0	2.4248
F5GYV5;H7BZV4;A0A0C4DG62;F8WCT1;Q66PJ3- 7;Q66PJ3-4;Q66PJ3-3;Q66PJ3- 2;Q66PJ3;F8WEP2;A0A0C4DH18;F5H7X4;Q66PJ3-	ADP-ribosylation factor-like protein 6-interacting protein 4	ARL6IP4	13	2	2	2	13.3	23.687	0	47.009
Q92747;E9PF58;Q92747-2	Actin-related protein 2/3 complex subunit 1A	ARPC1A	4	4	4	3	21.1	41.569	0	15.113
O15143;C9JM51;F8WEB3;C9JTT6;C9JBJ7;C9K057; C9J4Z7;F8VXW2;C9JEY1;C9JQM8;C9JFG9;C9J6C8	Actin-related protein 2/3 complex subunit 1B	ARPC1B	12	2	1	1	10.8	40.949	0.008375	1.7435
O15144	Actin-related protein 2/3 complex subunit 2	ARPC2	4	5	5	5	18.3	34.333	0	7.6153
F8VR50;C9JZD1;O15145	Actin-related protein 2/3 complex subunit 3	ARPC3	3	1	1	1	13.1	9.7261	0.008292	1.7294
F8WDD7;F8WCF6;A0A0A6YYG9;P59998;P59998- 2;F8WDW3;F8WE39;R4GN08;P59998-4;P59998-3	Actin-related protein 2/3 complex subunit 4	ARPC4;ARPC4-TTLL3	10	2	2	2	17.7	13.057	0.005505	2.274
H0Y2W2;Q9NVI7-3;Q9NVI7-2;Q9NVI7;Q5T9A4- 3;Q5T9A4	ATPase family AAA domain-containing protein 3A;ATPase family AAA domain-containing protein 3B	ATAD3A;ATAD3B	9	7	7	7	14.9	64.243	0	14.38
P05023-3;P05023-4;P05023;P05023- 2;M0R116;B1AKY9;A0A0A0MT26;P13637;P50993; P13637-2;P13637-3	Sodium/potassium-transporting ATPase subunit alpha- 1;Sodium/potassium-transporting ATPase subunit alpha- 3;Sodium/potassium-transporting ATPase subunit alpha-	ATP1A1;ATP1A3;ATP 1A2	14	4	4	4	6.1	109.55	0	8.1428
P16615;P16615-5;P16615-2;P16615-3;P16615-	Sarcoplasmic/endoplasmic reticulum calcium ATPase 2	ATP2A2	18	12	12	12	16.9	114.76	0	56.792
P25705-	ATP synthase subunit alpha, mitochondrial	ATP5A1	9	5	5	5	13.1	54.493	0	24.595
F8W0P7;H0YH81;P06576	ATP synthase subunit beta;ATP synthase subunit beta,	ATP5B	3	2	2	2	10	28.392	0	2.9108
P36542-2;P36542	ATP synthase subunit gamma, mitochondrial	ATP5C1	2	3	3	3	18.2	32.881	0	20.894
P56134-3;P56134;C9JJT5;C9JU26;G3V325;P56134- 4;P56134-2	ATP synthase subunit f, mitochondrial	ATP5J2;ATP5J2- PTCD1	7	3	3	3	67.3	6.2953	0	15.506
Q8WWM7-6;Q8WWM7-8;Q8WWM7-5;Q8WWM7- 4;Q8WWM7-9;Q8WWM7-2;Q8WWM7;Q8WWM7-	Ataxin-2-like protein	ATXN2L	9	2	2	2	3.3	102.89	0	3.6621
Q9Y679-2;Q9Y679;Q9Y679-3	Ancient ubiquitous protein 1	AUP1	3	3	3	3	14.1	45.786	0	9.3866
O95816;O95816-2	BAG family molecular chaperone regulator 2	BAG2	2	10	10	10	57.3	23.772	0	323.31
F6S6P2;X6REW1;P46379-4;P46379-5;P46379- 2;P46379;P46379- 3;F6TC96;F6UR09;F6U1F2;F6XTU0;A0A0A6YYP0;F	Large proline-rich protein BAG6	BAG6	16	2	2	2	8.5	54.67	0.008621	1.8961
O75531	Barrier-to-autointegration factor;Barrier-to- autointegration factor, N-terminally processed	BANF1	1	2	2	2	40.4	10.058	0	8.5608
Q9UIG0-2;Q9UIG0	Tyrosine-protein kinase BAZ1B	BAZ1B	2	3	3	3	3.6	170.45	0	2.9032
O75934	Pre-mRNA-splicing factor SPF27	BCAS2	1	2	2	2	18.2	26.131	0	8.6927
Q9NYF8-2;Q9NYF8;Q9NYF8-4	Bcl-2-associated transcription factor 1	BCLAF1	4	32	32	3	33.9	105.95	0	323.31
E9PK91;Q9NYF8-3;E9PK09;E9PQN2;E9PKI6	Bcl-2-associated transcription factor 1	BCLAF1	8	29	1	1	34	100.4	0	89.503
Q14137;Q14137-2	Ribosome biogenesis protein BOP1	BOP1	2	3	3	3	8.4	83.629	0	10.915
Q8NFQ5	BPI fold-containing family B member 6	BPIFB6	1	1	1	1	3.1	49.716	0	3.1798
C9JCD9;C9JNV2;P41223;P41223-2	Protein BUD31 homolog	BUD31	4	1	1	1	10.7	11.905	0.008065	1.611
H0YJB9;G3V4C6;Q9Y224	UPF0568 protein C14orf166	C14orf166	3	1	1	1	12.4	14.726	0.005515	2.2776
Q07021;I3L3Q7;I3L3B0	Complement component 1 Q subcomponent-binding protein, mitochondrial	C1QBP	3	4	4	4	26.2	31.362	0	17.572
P0C0L4;P0C0L4-2;P0C0L5;F5GXS0	Complement C4-A;Complement C4 beta chain;Complement C4-A alpha chain;C4a anaphylatoxin;C4b-A;C4d-A;Complement C4 gamma chain;Complement C4-B;Complement C4 beta chain;Complement C4-B alpha chain;C4a	C4A;C4B	6	4	4	4	3.7	192.78	0	7.2263
A0A0A6YYC3;Q96HJ9-2;A0A0A6YYJ8;Q9Y383- 3;Q9Y383-2;Q9Y383	Putative RNA-binding protein Luc7-like 2	C7orf55- LUC7L2;LUC7L2	11	3	3	3	12.3	46.228	0	19.818
H0Y7A7;P62158;E7ETZ0;E7EMB3;G3V361;Q96HY	Calmodulin	CALM2;CALM1	10	5	5	5	31.6	20.762	0	323.31
Q9NZT1	Calmodulin-like protein 5	CALML5	1	1	1	1	9.6	15.892	0.008681	1.9273
B1AKR1		CALML6	1	1	1	1	6.7	18.736	0.00936	1.4343
P27797;K7EJB9	Calreticulin	CALR	3	5	5	5	21.8	48.141	0	8.1598
P27824;P27824-2;P27824-3;D6RGY2	Calnexin	CANX	11	10	10	10	30.2	67.567	0	38.753
P52907	F-actin-capping protein subunit alpha-1	CAPZA1	1	11	11	9	65.7	32.922	0	187.61
P47755;C9JUG7;F8W9N7;P47755-2	F-actin-capping protein subunit alpha-2	CAPZA2	5	6	4	4	30.8	32.949	0	19.342
B1AK87;B1AK88;P47756-2;B1AK85;P47756	F-actin-capping protein subunit beta	CAPZB	5	7	7	7	38.1	29.295	0	106.86
E7ENE0;P41180;P41180-2	Extracellular calcium-sensing receptor	CASR	3	52	52	52	53.7	121.77	0	323.31
Q13185;C9JMM0;B8ZZ43;S4R2Y4	Chromobox protein homolog 3	CBX3	5	4	4	4	20.8	20.811	0	11.595
P50991-2;P50991	T-complex protein 1 subunit delta	CCT4	2	1	1	1	2.6	54.719	0.009539	1.4862
Q99459	Cell division cycle 5-like protein	CDC5L	1	12	12	12	26.7	92.25	0	43.979
B5MBX0;Q96FF9	Sororin	CDCA5	2	1	1	1	2.9	33.734	0.005566	2.2982
H7BY79;Q8IXH8	Cadherin-like protein 26	CDH26	2	1	1	1	2.5	48.924	0	2.4721
Q5VV42-2;Q5VV42	Threonylcarbamoyladenosine tRNA	CDKAL1	2	4	4	4	13.7	54.666	0	11.106
A0A0B4J291;Q8N8U2	Chromodomain Y-like protein 2	CDYL2	2	1	1	1	3.9	56.557	0.009524	1.4836
E9PP50;P23528;E9PLJ3;E9PS23;E9PQB7;G3V1A4; E9PK25;Q9Y281-3;Q9Y281	Cofilin-1;Cofilin-2	CFL1;CFL2	9	2	2	2	14.5	17.777	0	3.681
Q8N4Q1;Q8N4Q1-2	Mitochondrial intermembrane space import and	CHCHD4	2	1	1	1	16.9	15.996	0	3.167
O14646-2;O14646;H0Y8V4;A0A087WVF4;O14647- 2;O14647	Chromodomain-helicase-DNA-binding protein 1;Chromodomain-helicase-DNA-binding protein 2	CHD1;CHD2	6	2	2	2	2.5	196.59	0	4.1798
Q8IWX8;J3QK89	Calcium homeostasis endoplasmic reticulum protein	CHERP	2	13	13	13	22.9	103.7	0	96.872
X6R700;Q9Y3Y2-4;Q9Y3Y2;Q9Y3Y2-3	Chromatin target of PRMT1 protein	CHTOP	6	3	3	3	22	23.661	0	6.8049
Q8N5K1;I3L1N9;D6RCF4	CDGSH iron-sulfur domain-containing protein 2	CISD2	3	2	2	2	16.3	15.278	0.007169	2.1559
Q07065	Cytoskeleton-associated protein 4	CKAP4	1	2	2	2	5.5	66.022	0	3.0819
A0A087WVQ6;Q00610-2;Q00610	Clathrin heavy chain;Clathrin heavy chain 1	CLTC	8	12	12	12	9.6	192.06	0	28.145
A6NLH6;Q9P003	Protein cornichon homolog 4	CNIH4	2	1	1	1	14.6	15.771	0.009331	1.4293
Q9P232	Contactin-3	CNTN3	1	1	1	1	1.4	112.88	0.009554	1.4966
Q8WYK1	Contactin-associated protein-like 5	CNTNAP5	1	1	1	1	1.6	145.62	0.008117	1.6335
P38432	Coilin	COIL	1	2	2	2	5.2	62.608	0	2.8723
P21964-2;P21964	Catechol O-methyltransferase	COMT	2	1	1	1	10	24.449	0	3.3068
Q10570	Cleavage and polyadenylation specificity factor subunit 1	CPSF1	1	6	6	6	5.7	160.88	0	9.3615
Q9P2I0;H0YJF4	Cleavage and polyadenylation specificity factor subunit 2	CPSF2	2	4	4	4	7	88.486	0	6.3452
Q5JY65;Q9BZJ0-2;Q9BZJ0-3;Q9BZJ0	Crooked neck-like protein 1	CRNKL1	4	1	1	1	1.4	99.172	0	2.4536
P55060-4;P55060-3;P55060	Exportin-2	CSE1L	3	2	2	2	2.6	103.88	0	3.0339
E7EU96;Q5U5J2;Q8NEV1;P68400	Casein kinase II subunit alpha 3;Casein kinase II subunit	CSNK2A1;CSNK2A3	8	5	5	5	24.4	45.31	0	12.853
P19784;H3BSA1;H3BV19	Casein kinase II subunit alpha	CSNK2A2	4	9	9	9	38.9	41.213	0	21.173
Q5SRQ3;Q5SRQ6;N0E472;P67870;N0E644	Casein kinase II subunit beta	CSNK2B-LY6G5B- 1181;CSNK2B;CSNK2 B-LY6G5B-- 991;CSNK2B-LY6G5B- 562	5	3	3	3	18	26.673	0	12.084
Q14247-3;Q14247;Q14247-2;H7C314	Src substrate cortactin	CTTN	4	2	2	2	8.8	57.466	0.008562	1.8208
Q16643;Q16643-3;Q16643-2;D6R9W4	Drebrin	DBN1	7	19	19	19	47.5	71.428	0	323.31
Q96PD2;Q96PD2-2	Discoidin, CUB and LCCL domain-containing protein 2	DCBLD2	2	1	1	1	1.5	85.034	0.009231	1.3894
A0A0C4DGS1;P39656-2;P39656;P39656-3	Dolichyl-diphosphooligosaccharide--protein glycosyltransferase 48 kDa subunit	DDOST	5	5	5	5	21	48.799	0	23.016
F1T0B3;A0A087X2G1;Q92499-3;Q92499;Q92499-	ATP-dependent RNA helicase DDX1	DDX1	5	3	3	3	7.3	73.915	0	5.9769
H3BLZ8;Q92841-1;Q92841-3;Q92841-2;Q92841	Probable ATP-dependent RNA helicase DDX17	DDX17	5	16	11	11	26.4	80.439	0	43.573
Q9NVP1	ATP-dependent RNA helicase DDX18	DDX18	2	6	6	6	13.9	75.406	0	7.3026
Q9UHI6	Probable ATP-dependent RNA helicase DDX20	DDX20	2	3	3	3	5.5	92.239	0	2.6981
Q9NR30;Q9NR30-2	Nucleolar RNA helicase 2	DDX21	2	17	17	15	30.5	87.343	0	77.406
Q13838;Q13838- 2;Q5STU3;O00148;F6QYI9;F6R6M7;F6S4E6;A0A0 A0MT12;F6TRA5;F6WLT2	Spliceosome RNA helicase DDX39B;ATP-dependent RNA helicase DDX39A	DDX39B;DDX39A	25	7	7	7	19.9	48.991	0	36.766
O00571;O00571-2;O15523-2;O15523	ATP-dependent RNA helicase DDX3X;ATP-dependent RNA helicase DDX3Y	DDX3X;DDX3Y	15	19	19	18	43.4	73.243	0	323.31
J3KNN5;Q9UJV9	Probable ATP-dependent RNA helicase DDX41	DDX41	5	8	8	8	15.5	71.648	0	12.162

A0A0C4DG89;Q7L014	Probable ATP-dependent RNA helicase DDX46	DDX46	2	2	2	2	2.3	117.46	0.009317	1.4265
J3KTA4;P17844;P17844-2	Probable ATP-dependent RNA helicase DDX5	DDX5	12	19	18	14	37.5	69.086	0	150.15
A0A087WVC1;A0A087WVB0;Q9BQ39	ATP-dependent RNA helicase DDX50	DDX50	3	5	3	3	9.6	82.226	0	7.097
F8WDT8;G3V0G3;H7C3E9;Q9NY93-2;Q9NY93	Probable ATP-dependent RNA helicase DDX56	DDX56	5	1	1	1	7.6	20.342	0.009419	1.4444
P26196;Q8IV96	Probable ATP-dependent RNA helicase DDX6	DDX6	2	2	2	2	7	54.416	0.001866	2.3186
H0Y993;B4DFG0;P35659-2;P35659	Protein DEK	DEK	4	1	1	1	8.3	17.78	0.00832	1.7326
I3L112;P52429	Diacylglycerol kinase;Diacylglycerol kinase epsilon	DGKE	2	1	1	1	2.9	51.101	0	2.7338
O43143	Pre-mRNA-splicing factor ATP-dependent RNA helicase	DHX15	4	29	29	29	46.4	90.932	0	295.21
H7BXY3;Q7L2E3-3;Q7L2E3;Q7L2E3-2	Putative ATP-dependent RNA helicase DHX30	DHX30	4	14	14	14	16.8	130.55	0	26.717
Q08211	ATP-dependent RNA helicase A	DHX9	2	18	18	18	22.8	140.96	0	163.14
Q96QB1-4;Q96QB1-1;Q96QB1-6;Q96QB1	Rho GTPase-activating protein 7	DLC1	4	1	1	1	2.6	114.15	0.009434	1.4456
P31689;P31689-2	DnaJ homolog subfamily A member 1	DNAJA1	2	2	2	2	6.5	44.868	0	3.4783
H7C2Y5;Q9UBS4	DnaJ homolog subfamily B member 11	DNAJB11	2	1	1	1	6.6	19.149	0	5.7773
O75165	DnaJ homolog subfamily C member 13	DNAJC13	1	6	6	6	5.1	254.41	0	7.6092
Q99615;Q99615-2;K7ESP1;K7EIH8;K7EPP7	DnaJ homolog subfamily C member 7	DNAJC7	13	18	18	18	41.3	56.44	0	154.39
K7ESB7;Q96HP0	Dedicator of cytokinesis protein 6	DOCK6	2	1	1	1	1.2	148.68	0.007975	1.5136
Q96N67-4;Q96N67-3;Q96N67-5;Q96N67-	Dedicator of cytokinesis protein 7	DOCK7	9	21	21	21	15.8	238.27	0	115.33
Q5QPK2;H0Y368;O60762;Q5QPJ9	Dolichol-phosphate mannosyltransferase subunit 1	DPM1	4	4	4	4	20.9	32.426	0	10.266
E5RFU4;Q16555-2;Q16555	Dihydropyrimidinase-related protein 2	DPYSL2	3	1	1	1	11.2	18.331	0.005525	2.2861
Q14126	Desmoglein-2	DSG2	1	3	3	3	6.4	122.29	0	3.4326
P15924-2;P15924-3;P15924	Desmoplakin	DSP	3	10	10	10	7.4	260.12	0	24.502
B4DWJ3;Q96CJ1	ELL-associated factor 2	EAF2	2	1	1	1	4.6	29.172	0.007273	2.2118
H7C2Q8;Q99848	Probable rRNA-processing protein EBP2	EBNA1BP2	2	2	2	2	5.5	40.684	0.007143	2.1289
P68104;A0A087WVQ9;P68104-2;Q5VTE0;A0A087WV01	Elongation factor 1-alpha 1;Putative elongation factor 1-alpha-like 3;Elongation factor 1-alpha	EEF1A1;EEF1A1P5	8	11	11	11	45.5	50.14	0	167.5
P26641;P26641-2	Elongation factor 1-gamma	EEF1G	2	4	4	4	9.8	50.118	0	6.2861
P13639	Elongation factor 2	EEF2	1	4	4	4	5.6	95.337	0	5.8268
Q15029-2;Q15029;Q15029-3	116 kDa U5 small nuclear ribonucleoprotein component	EFTUD2	5	14	14	14	21.9	105.38	0	50.712
P60842;J3QKZ9;J3QLN6;J3KS25;J3QR64;J3KTB5;J3QL43;J3QS69;J3KT12;E7EQG2;P60842-2;Q14240;Q14240-	Eukaryotic initiation factor 4A-I;Eukaryotic initiation factor 4A-II;Eukaryotic initiation factor 4A-II, N-terminally processed	EIF4A1;EIF4A2	17	4	3	3	12.6	46.153	0	5.4432
P38919	Eukaryotic initiation factor 4A-III;Eukaryotic initiation factor 4A-III, N-terminally processed	EIF4A3	5	15	15	14	46.2	46.871	0	64.206
E7EX17;P23588;P23588-2;F8W0K0;F8VSC7;F8VP89;F8VX11;F8VYE9	Eukaryotic translation initiation factor 4B	EIF4B	9	9	9	9	21.1	69.697	0	181.82
F8WD20;B7ZBH1;A0A0B4J1Y7;F8WDS6;P56537-	Eukaryotic translation initiation factor 6	EIF6	6	1	1	1	23.7	7.005	0.00708	2.0868
Q15717;Q15717-2;M0QZR9	ELAV-like protein 1	ELAVL1	4	8	8	8	35.6	36.091	0	17.519
P50402;Q5HY57	Emerin	EMD	2	3	3	3	18.1	28.994	0	4.951
P58107	Epiplakin	EPPK1	1	14	11	1	22.4	555.61	0	227.63
P84090;G3V279	Enhancer of rudimentary homolog	ERH	2	6	6	6	76	12.259	0	323.31
O75477	Erlin-1	ERLIN1	1	3	1	1	11	38.925	0	2.8497
E5RHW4;O94905	Erlin-2	ERLIN2	6	11	11	9	47	37.725	0	100.96
Q9BSJ8;Q9BSJ8-2	Extended synaptotagmin-1	ESYT1	3	5	5	5	5.8	122.85	0	8.9002
Q96CS3	FAS-associated factor 2	FAF2	2	6	6	6	22.7	52.623	0	13.177
E9PH82;Q8NCA5-2;Q8NCA5	Protein FAM98A	FAM98A	3	1	1	1	4.8	34.431	0.005495	2.2482
P22087;M0R299;M0QXL5;M0R2Q4;M0R0P1;M0R2U2;M0R1H0;M0R2B0	rRNA 2-O-methyltransferase fibrillarin	FBL	8	8	8	7	38	33.784	0	18.722
C9JI88;A0A0A0MRW5;E7ERH8;Q8NEE6-4;Q8NEE6-3;Q8NEE6-2;Q8NEE6	F-box/LRR-repeat protein 13	FBXL13	7	1	1	1	5.2	25.101	0.008333	1.7332
E9PNM1;P37268-4;P37268-3;P37268-2;P37268-	Squalene synthase	FDFT1	6	1	1	1	4.4	47.285	0	3.7403
A0A087WV25;O95684-2;O95684;O95684-3	FGFR1 oncogene partner	FGFR1OP	4	2	2	2	12	38.099	0	5.0106
Q6UN15-3;Q6UN15-5;Q6UN15;Q6UN15-	Pre-mRNA 3-end-processing factor FIP1	FIP1L1	6	7	7	7	23.3	58.375	0	17.242
Q14318;Q14318-2;U3KQ64;M0R2K9	Peptidyl-prolyl cis-trans isomerase FKBP8;Peptidyl-prolyl cis-trans isomerase	FKBP8	7	3	3	3	10.9	44.561	0	3.8149
Q5HY54;Q60FE5;P21333-2;P21333;A0A087WWY3	Filamin-A	FLNA	11	37	37	36	24.7	276.55	0	185.77
O75369-6;O75369-3;O75369-2;O75369-9;O75369;O75369-8;E7EN95;O75369-5;O75369-	Filamin-B	FLNB	10	3	2	2	2.6	271.41	0	4.3658
A2AB09;O75955-2;O75955	Flotillin-1	FLOT1	3	1	1	1	5.5	27.327	0.008576	1.8224
E9PD35;P35916-1;P35916	Receptor protein-tyrosine kinase;Vascular endothelial growth factor receptor 3	FLT4	3	1	1	1	1	146.45	0.009404	1.4437
Q06787-7;Q06787;Q06787-6;Q06787-4;Q06787-5;Q06787-3;G8JLE9	Fragile X mental retardation protein 1	FMR1	7	17	15	1	40.5	69.178	0	246.47
Q8IY81	pre-rRNA processing protein FTSJ3	FTSJ3	1	4	4	4	7.6	96.557	0	5.6624
J3KSP6		FUK	1	1	1	1	2.3	62.219	0.009245	1.3916
P51114;P51114-2;B4DXZ6;E9PFF5;P51114-	Fragile X mental retardation syndrome-related protein 1	FXR1	13	24	24	23	50.9	69.72	0	323.31
P51116	Fragile X mental retardation syndrome-related protein 2	FXR2	2	20	19	18	47.8	74.222	0	323.31
Q14697;Q14697-2;F5H6X6;E9PKU7	Neutral alpha-glucosidase AB	GANAB	5	19	19	19	31.2	106.87	0	52.609
P04406-2;P04406;E7EUT5	Glyceraldehyde-3-phosphate dehydrogenase	GAPDH	3	4	4	4	23.9	31.548	0	11.925
Q9NY12-2;Q9NY12	H/ACA ribonucleoprotein complex subunit 1	GAR1	2	1	1	1	9	20.834	0.007299	2.2191
C9JH18;I1E4Y6;Q6Y7W6-4;Q6Y7W6-	PERQ amino acid-rich with GYF domain-containing	GIGYF2	6	1	1	1	10.1	16.78	0.009288	1.4258
P08754;P63096;P04899;P04899-4;P63096-2;P04899-6;P04899-3;P04899-5;P04899-2;P09471-2	Guanine nucleotide-binding protein G(k) subunit alpha;Guanine nucleotide-binding protein G(i) subunit alpha-1;Guanine nucleotide-binding protein G(i) subunit alpha-2;Guanine nucleotide-binding protein G(o) subunit	GNAI3;GNAI1;GNAI2;GNAO1	31	5	5	5	16.9	40.532	0	51.624
B1AKQ8;F6X3N5;F6UT28;P62873-2;P62873	Guanine nucleotide-binding protein G(I)/G(S)/G(T)	GNB1	5	3	1	1	33.6	12.198	0	14.459
C9JXA5;P62879;C9JIS1;E7EP32;C9JZN1;P62879-2	Guanine nucleotide-binding protein G(I)/G(S)/G(T)	GNB2	8	6	6	4	39.4	27.561	0	9.423
D6RAC2;D6REE5;P63244;D6RGK8;H0Y9P0;D6RF23;D6R909;D6RAU2;E9PD14;D6RBD0;D6RFX4;D6RFZ9;H0YAF8;D6RHH4;D6R9Z1;H0YAM7;J3KPE3;H0Y8	Guanine nucleotide-binding protein subunit beta-2-like 1;Guanine nucleotide-binding protein subunit beta-2-like 1, N-terminally processed	GNB2L1	19	2	2	2	9.7	29.75	0	22.022
Q9BVP2-2;Q9BVP2	Guanine nucleotide-binding protein-like 3	GNL3	2	3	3	3	5.4	60.54	0	3.4761
Q8NBJ4-2;Q8NBJ4	Golgi membrane protein 1	GOLM1	2	1	1	1	4.6	44.272	0	3.249
Q9Y5Y3	Probable G-protein coupled receptor 45	GPR45	1	1	1	1	4	41.966	0	2.9954
Q9BZE4-3;Q9BZE4-2;Q9BZE4;Q5T3R7	Nucleolar GTP-binding protein 1	GTPBP4	4	2	2	2	5	60.051	0	2.7628
Q71UI9;P0C0S5;C9J0D1;Q71UI9-4;Q71UI9-	Histone H2A.V;Histone H2A.Z;Histone H2A	H2AFV;H2AFZ	7	4	2	2	46.1	13.509	0	117.1
P16104;Q96QV6;Q8IUE6	Histone H2AX;Histone H2A type 1-A;Histone H2A type 2-B	H2AFX;HIST1H2AA;HIST2H2AB	3	5	1	1	52.4	15.144	0	11.775
O75367-2;O75367-3;O75367;B4DJC3	Core histone macro-H2A.1;Histone H2A	H2AFY	7	9	9	9	35.2	39.183	0	40.468
K7EK07;P84243;K7EMV3;B4DEB1;K7ES00;K7EP01;	Histone H3;Histone H3.3;Histone H3.3C	H3F3B;H3F3A;H3F3C	7	4	1	1	44.7	14.914	0	70.722
H3BRL8;H3BPZ1;H3BS72;Q9P035-	Very-long-chain (3R)-3-hydroxyacyl-CoA dehydratase 3	HACD3	6	3	3	3	10.2	29.205	0	60.882
P40939;H0YFD6	Trifunctional enzyme subunit alpha, mitochondrial;Long-chain enoyl-CoA hydratase;Long chain 3-hydroxyacyl-CoA	HADHA	3	8	8	8	13.6	82.999	0	24.065
B5MD38;F5GZQ3;P55084-2;P55084	Trifunctional enzyme subunit beta, mitochondrial;3-ketoacyl-CoA thiolase	HADHB	4	1	1	1	3.7	37.924	0.008306	1.7299
P68871;F8W6P5	Hemoglobin subunit beta;LVV-hemorphin-7;Spinorphin	HBB	2	4	2	2	29.9	15.998	0	3.9079
Q9UQL6-2;Q9UQL6;Q9UQL6-3	Histone deacetylase 5	HDAC5	3	1	1	1	1.5	112.19	0.009302	1.4258
P16403	Histone H1.2	HIST1H1C	1	5	5	1	23	21.364	0	54.568
P10412;P16402	Histone H1.4;Histone H1.3	HIST1H1E;HIST1H1D	4	5	1	1	22.4	21.865	0	2.8287
Q99878;Q96KK5;Q9BTM1;P20671;P0C0S8;H0YFX9;Q93077;Q7L7L0;P04908;Q9BTM1-2	Histone H2A type 1-J;Histone H2A type 1-H;Histone H2A.J;Histone H2A type 1-D;Histone H2A type 1;Histone H2A;Histone H2A type 1-C;Histone H2A type 3;Histone H2A type 1-B/E	HIST1H2AJ;HIST1H2AH;H2AFJ;HIST1H2AD;HIST1H2AG;HIST1H2AC;HIST3H2A;HIST1H2AB	11	6	6	0	58.6	13.936	0	99.997
U3KQK0;Q99880;Q99879;Q99877;Q93079;Q5QNW6;P62807;P58876;P57053;O60814;Q5QNW6-2	Histone H2B;Histone H2B type 1-L;Histone H2B type 1-M;Histone H2B type 1-N;Histone H2B type 1-H;Histone H2B type 2-F;Histone H2B type 1-C/E/F/G/I;Histone H2B type 1-D;Histone H2B type F-S;Histone H2B type 1-K	HIST1H2BN;HIST1H2BL;HIST1H2BM;HIST1H2BH;HIST2H2BF;HIST1H2BC;HIST1H2BD;H2BFS;HIST1H2BK	12	4	2	2	20.5	18.804	0	28.986
P68431	Histone H3.1	HIST1H3A	1	4	1	1	43.4	15.404	0	38.691
P62805	Histone H4	HIST1H4A	1	7	7	7	43.7	11.367	0	254.87
Q16777;Q6FI13	Histone H2A type 2-C;Histone H2A type 2-A	HIST2H2AC;HIST2H2A A3	2	6	1	1	58.1	13.988	0	23.248
Q71DI3	Histone H3.2	HIST2H3A	1	4	4	1	43.4	15.388	0	93.761
Q5TEC6	Histone H3	HIST2H3PS2	1	3	1	1	19.9	15.43	0	34.454
Q8N257;Q16778;P33778;P23527;P06899;Q6DRA6;Q6DN03	Histone H2B type 3-B;Histone H2B type 2-E;Histone H2B type 1-B;Histone H2B type 1-O;Histone H2B type 1-J;Putative histone H2B type 2-D;Putative histone H2B type 2-C	HIST3H2BB;HIST2H2BE;HIST1H2BB;HIST1H2BO;HIST1H2BJ;HIST2H2BD;HIST2H2BC	7	4	4	2	27	13.908	0	179.24
I3L276;I3L4P8;A0A0A6YYS0;I3L463;I3L159;I3L1F5;	Heme oxygenase 2	HMOX2	7	1	1	1	14.5	12.647	0	3.0603
Q13151	Heterogeneous nuclear ribonucleoprotein A0	HNRNPA0	1	2	2	2	11.8	30.84	0	5.4317
F8W6I7;P09651-3;P09651-2;P09651;F8VZ49;Q32P51;F8VTQ5	Heterogeneous nuclear ribonucleoprotein A1;Heterogeneous nuclear ribonucleoprotein A1, N-terminally processed;Heterogeneous nuclear	HNRNPA1;HNRNPA1L2	10	7	7	7	25.1	33.155	0	36.629
P22626;A0A087WUI2;P22626-2	Heterogeneous nuclear ribonucleoproteins A2/B1	HNRNPA2B1	3	11	11	11	31.4	37.429	0	56.073
P51991;P51991-2	Heterogeneous nuclear ribonucleoprotein A3	HNRNPA3	3	8	8	8	25.4	39.594	0	124.9
D6R9P3;D6RD18;Q99729-		HNRNPAB	6	1	1	1	5	30.302	0	2.8377
G3V576;G3V2Q1;P07910;G3V555;G3V575	Heterogeneous nuclear ribonucleoproteins C1/C2	HNRNPC	5	8	1	1	43.3	25.256	0	26.511
G3V4W0;B4DY08;B2R5W2;G3V4C1;P07910-4;P07910-2;G3V5X6;G3V3K6;G3V251;B4DSU6;G3V2D6;P07910-3;P0DMR1;O60812;B7ZW38;B2RXH8	Heterogeneous nuclear ribonucleoproteins C1/C2;Heterogeneous nuclear ribonucleoprotein C-like 4;Heterogeneous nuclear ribonucleoprotein C-like 1;Heterogeneous nuclear ribonucleoprotein C-like	HNRNPC;HNRNPCL4;HNRNPCL1;HNRNPCL3;HNRNPCL2	19	8	8	1	33.2	28.916	0	120.84
D6RF44;H0YA96;D6RAF8;H0Y8G5;Q14103-4;Q14103-3;Q14103-2;Q14103;D6RBQ9	Heterogeneous nuclear ribonucleoprotein D0	HNRNPD	10	3	3	2	28.8	12.553	0	6.1136

P52597	Heterogeneous nuclear ribonucleoprotein F;Heterogeneous nuclear ribonucleoprotein F, N-	HNRNPF	1	4	2	2	18.1	45.671	0	55.475
G8JLB6;P31943;E9PCY7;H0YB39;D6RIT2;D6RIU0;D6RBM0	Heterogeneous nuclear ribonucleoprotein H;Heterogeneous nuclear ribonucleoprotein H, N-	HNRNPH1	24	10	10	8	32.8	51.229	0	323.31
P61978-3;P61978;P61978-2;Q5T6W2	Heterogeneous nuclear ribonucleoprotein K	HNRNPK	6	14	14	14	37.7	48.562	0	323.31
P14866;M0QXS5;P14866-2	Heterogeneous nuclear ribonucleoprotein L	HNRNPL	8	12	12	12	44.8	64.132	0	139.51
A0A087X0X3;P52272-2;P52272;M0R019	Heterogeneous nuclear ribonucleoprotein M	HNRNPM	10	15	15	10	34.8	77.569	0	134.36
M0QZM1		HNRNPM	1	6	1	1	31.9	39.933	0.008772	1.999
O43390;O43390-4;O43390-2;B4DT28;O43390-3	Heterogeneous nuclear ribonucleoprotein R	HNRNPR	5	7	7	5	16.3	70.942	0	28.121
Q00839-2;Q00839	Heterogeneous nuclear ribonucleoprotein U	HNRNPU	3	13	13	13	24.1	88.979	0	323.31
Q53GQ0;E9PI21	Very-long-chain 3-oxoacyl-CoA reductase	HSD17B12	2	2	2	2	8.3	34.324	0.008636	1.9056
P07900;P07900-2	Heat shock protein HSP 90-alpha	HSP90AA1	4	10	4	4	19	84.659	0	6.0007
P08238	Heat shock protein HSP 90-beta	HSP90AB1	4	15	15	7	25.8	83.263	0	116.11
P14625;Q96GW1	Endoplasmin	HSP90B1	4	4	3	3	5.4	92.468	0	3.2049
P08107;P08107-2;V9GZ37	Heat shock 70 kDa protein 1A	HSPA1A	3	22	21	12	47.6	70.051	0	323.31
P34931	Heat shock 70 kDa protein 1-like	HSPA1L	1	9	1	1	22.5	70.374	0.008251	1.7205
P11021	78 kDa glucose-regulated protein	HSPA5	1	23	22	22	39.8	72.332	0	323.31
P17066;P48741	Heat shock 70 kDa protein 6;Putative heat shock 70 kDa	HSPA6;HSPA7	2	7	1	1	12	71.027	0	2.7696
P11142;E9PKE3;P11142-2;E9PNE6;E9PN89	Heat shock cognate 71 kDa protein	HSPA8	16	25	25	22	45.4	70.897	0	323.31
P38646	Stress-70 protein, mitochondrial	HSPA9	5	18	18	18	34.2	73.68	0	315.77
P10809	60 kDa heat shock protein, mitochondrial	HSPD1	7	6	6	6	16.2	61.054	0	34.538
Q9NZI8;Q9NZI8-2	Insulin-like growth factor 2 mRNA-binding protein 1	IGF2BP1	2	11	11	10	23.4	63.48	0	145.71
F8W930;Q9Y6M1-5;Q9Y6M1-6;Q9Y6M1-3;Q9Y6M1-4;Q9Y6M1-1;Q9Y6M1	Insulin-like growth factor 2 mRNA-binding protein 2	IGF2BP2	7	3	2	2	8.3	66.785	0	3.4669
O00425	Insulin-like growth factor 2 mRNA-binding protein 3	IGF2BP3	3	7	6	6	18.7	63.704	0	15.93
P01859	Ig gamma-2 chain C region	IGHG2	1	1	1	1	2.8	35.9	0	11.465
A0A087X130;A0A087WZW8;A0A087WYL9;A0A087WWV8;A0A087WTX5;P01834;A0A075B6H6	Ig kappa chain C region	IGKC;IGKV3-11;IGKV1-8	7	5	5	5	29.9	25.136	0	59.733
A0A087X0P6;A0A0A0MRZ7;A0A075B6S2		IGKV2D-29;IGKV2D-26	14	3	3	2	19.6	11.192	0	162.67
A0A075B6L1;A0A075B6L0;A0A075B6K9;P0CG06;P0CG05;P0CF74;A0M8Q6;A0A075B6K8;A0A0B4J231;A0A087WWC9;A0A087WXC3;A0A087WWU7;A0A087WYN7;A0A087WWT5;A0A087WU42;A0A087	Ig lambda-3 chain C regions;Ig lambda-2 chain C regions;Ig lambda-6 chain C region;Ig lambda-7 chain C region;Ig lambda-1 chain C regions;Immunoglobulin lambda-like polypeptide 5	IGLC7;IGLC3;IGLC2;IGLC6;IGLC1;IGLL5	20	2	2	2	32.1	11.196	0	63.788
B4DY09;Q12905	Interleukin enhancer-binding factor 2	ILF2	4	9	9	9	40.9	38.91	0	87.321
Q12906;Q12906-5;Q12906-2;Q12906-3	Interleukin enhancer-binding factor 3	ILF3	17	14	14	1	29.6	95.337	0	74.604
Q12906-7;Q12906-4;Q12906-6	Interleukin enhancer-binding factor 3	ILF3	3	14	1	1	29.1	95.807	0.007105	2.1117
H7C463;C9J406;B9A067;Q16891-3;Q16891-	MICOS complex subunit MIC60	IMMT	7	2	2	2	5.9	68.064	0	3.7592
O14654	Insulin receptor substrate 4	IRS4	1	25	25	25	32.6	133.77	0	208.71
Q9ULR0;Q9ULR0-2;Q9ULR0-1	Pre-mRNA-splicing factor ISY1 homolog	ISY1	3	1	1	1	4.9	32.992	0.008039	1.5855
Q14573	Inositol 1,4,5-trisphosphate receptor type 3	ITPR3	1	1	1	1	1	304.1	0	2.5471
Q9HDC5	Junctophilin-1	JPH1	1	5	5	5	13.5	71.685	0	7.3648
P14923	Junction plakoglobin	JUP	5	8	8	8	16.4	81.744	0	16.955
Q14681	BTB/POZ domain-containing protein KCTD2	KCTD2	2	4	4	4	27	28.527	0	209.54
Q9NXV2;H3BSS5	BTB/POZ domain-containing protein KCTD5	KCTD5	2	7	7	7	53.8	26.092	0	238.69
P24390-2;P24390	ER lumen protein-retaining receptor 1	KDELR1	2	1	1	1	12.7	17.486	0.007194	2.1666
H7BYF7;P33947-2;P33947	ER lumen protein-retaining receptor 2	KDELR2	3	1	1	1	44.7	4.127	0.008696	1.9315
Q07666-3;Q07666-2;Q07666	KH domain-containing, RNA-binding, signal transduction-associated protein 1	KHDRBS1	3	2	2	2	8.4	44.027	0	10.748
M0QYH3;A0A087WTP3;Q92945;M0QXW7;M0R0I	Far upstream element-binding protein 2	KHSRP	5	3	3	3	26	18.017	0	6.1173
Q69YN4-3;Q69YN4	Protein virilizer homolog	KIAA1429	5	3	3	3	2.5	201.05	0	5.4767
Q9ULH0-5;Q9ULH0-2;Q9ULH0-4;Q9ULH0	Kinase D-interacting substrate of 220 kDa	KIDINS220	4	1	1	1	4.4	59.966	0.005556	2.2968
P52732	Kinesin-like protein KIF11	KIF11	1	50	50	50	56.6	119.16	0	323.31
P52292;J3QLL0;J3KS65	Importin subunit alpha-1	KPNA2	3	5	5	5	16.4	57.861	0	12.706
Q14974;Q14974-2;J3KTM9	Importin subunit beta-1	KPNB1	5	7	7	7	11.8	97.169	0	29.502
H0YFD2;F8WFC1;Q8N9T8	Protein KRI1 homolog	KRI1	3	1	1	1	7.4	22.2	0.009494	1.4673
F8VZY9;P05783	Keratin, type I cytoskeletal 18	KRT18	3	6	6	3	20.5	43.774	0	20.836
E5RH50;A0A0B4J210;Q6PKG0-3;Q6PKG0;H0YC33;H0YC73;H0YBW1	La-related protein 1	LARP1	7	2	2	2	4.6	69.746	0	3.5559
Q14739	Lamin-B receptor	LBR	2	3	3	3	6.2	70.702	0	7.8825
Q86U70-3;Q86U70-2;Q86U70	LIM domain-binding protein 1	LDB1	3	1	1	1	7	36.427	0.009274	1.4119
P47929	Galectin-7	LGALS7	1	2	2	2	18.4	15.075	0	5.0935
Q9UHB6;Q9UHB6-4;F8VQE1;Q9UHB6-2;Q9UHB6-5;F8VS07;Q9UHB6-3	LIM domain and actin-binding protein 1	LIMA1	10	24	24	24	40.3	85.225	0	323.31
P49257	Protein ERGIC-53	LMAN1	1	1	1	1	5.5	57.548	0	15.575
E9PBF6;P20700	Lamin-B1	LMNB1	2	2	2	2	5.7	44.643	0	2.4068
Q8NF37	Lysophosphatidylcholine acyltransferase 1	LPCAT1	1	1	1	1	2.8	59.151	0	3.2413
Q5VUJ6-2;Q5VUJ6	Leucine-rich repeat and calponin homology domain-containing protein 2	LRCH2	2	1	1	1	1.5	82.809	0	2.6807
Q96II8-3;Q96II8-4;Q96II8;Q96II8-2;E9PD99	Leucine-rich repeat and calponin homology domain-containing protein 3	LRCH3	7	6	6	6	15.2	78.801	0	22.645
Q96AG4	Leucine-rich repeat-containing protein 59	LRRC59	1	5	5	5	19.9	34.93	0	10.485
Q8IWT6	Volume-regulated anion channel subunit LRRC8A	LRRC8A	1	1	1	1	1.9	94.198	0.007246	2.1999
Q3MHD2;Q3MHD2-2;K7ELG9	Protein LSM12 homolog	LSM12	3	2	2	2	13.8	21.701	0.008651	1.9158
D6RDI2;J3KPP4;O95232;D6RHH0;U3KQT3	Luc7-like protein 3	LUC7L3	5	2	2	2	15.8	20.677	0	22.208
P61326;Q96A72;P61326-2;F5H6P7;F5H6N1	Protein mago nashi homolog;Protein mago nashi	MAGOH;MAGOHB	6	6	6	6	43.8	17.163	0	61.868
A8MXP9;P43243;D6REM6;D6R991;H0Y8T4	Matrin-3	MATR3	21	17	17	17	31.8	99.966	0	323.31
P25205;P25205-2;J3KQ69	DNA replication licensing factor MCM3	MCM3	4	4	4	4	6.7	90.98	0	4.7543
Q14676-2;Q14676;Q14676-3	Mediator of DNA damage checkpoint protein 1	MDC1	4	5	5	5	7.2	197.6	0	28.727
Q8IWI9-3;Q8IWI9;Q8IWI9-4	MAX gene-associated protein	MGA	3	1	1	1	0.5	315.22	0.00813	1.6458
Q99735-2;Q99735	Microsomal glutathione S-transferase 2	MGST2	2	1	1	1	18.2	8.3587	0	2.5691
A0A0A0MRH6;Q5JRA6-4;Q5JRA6-2;Q5JRA6	Melanoma inhibitory activity protein 3	MIA3	4	1	1	1	1.2	113.9	0.008475	1.7901
A0A087WV66;P46013-2;P46013	Antigen KI-67	MKI67	3	19	19	19	10.2	358.62	0	29.049
Q8N4V1;Q8N4V1-2	Membrane magnesium transporter 1	MMGT1	2	1	1	1	18.3	14.686	0	5.5963
Q9BU76-4;Q9BU76;Q9BU76-3;Q9BU76-2	Multiple myeloma tumor-associated protein 2	MMTAG2	4	4	4	4	23.8	20.536	0	6.9174
Q5JR04;Q9HCE1-2;Q9HCE1	Putative helicase MOV-10	MOV10	3	2	2	2	3.4	107.21	0.008143	1.6505
Q6WCQ1-3;Q6WCQ1;Q6WCQ1-2;J3KSW8;H0Y2S9	Myosin phosphatase Rho-interacting protein	MPRIP	7	6	6	6	10.2	114.05	0	31.726
H7C2P7;A6NJD9;A8MVT4;A8MYK1;Q16540	39S ribosomal protein L23, mitochondrial	MRPL23	5	1	1	1	12.7	13.669	0	14.188
E9PE17;I3L0E3;Q9Y2R5	28S ribosomal protein S17, mitochondrial	MRPS17;hCG_1984214	3	1	1	1	22.5	14.374	0.008026	1.5447
Q9Y676	28S ribosomal protein S18b, mitochondrial	MRPS18B	1	1	1	1	3.9	29.395	0.008711	1.9806
A0A075B746;P82921	28S ribosomal protein S21, mitochondrial	MRPS21	2	1	1	1	16.1	10.688	0	2.4312
E7EPW2;P82663-3;P82663-2;P82663	28S ribosomal protein S25, mitochondrial	MRPS25	4	2	2	2	27.3	12.946	0	3.9736
J3QQS1;J3QLS3;Q9Y2R9;J3KSI8;J3QKW2	28S ribosomal protein S7, mitochondrial	MRPS7	5	3	3	3	19.8	19.903	0	3.6606
P82933	28S ribosomal protein S9, mitochondrial	MRPS9	1	1	1	1	4	45.834	0	13.96
Q9UKD2	mRNA turnover protein 4 homolog	MRTO4	1	1	1	1	4.2	27.56	0.009509	1.478
P00846	ATP synthase subunit a	MT-ATP6	1	2	2	2	10.2	24.817	0.00846	1.7838
P03928	ATP synthase protein 8	MT-ATP8	1	1	1	1	16.2	7.9916	0.008265	1.7252
P84157-2;P84157-3;P84157	Matrix-remodeling-associated protein 7	MXRA7	3	1	1	1	17.1	17.493	0	4.6873
Q9BQG0;Q9BQG0-2;I3L1L3	Myb-binding protein 1A	MYBBP1A	3	7	7	7	8.7	148.85	0	17.803
A0A087WV05;Q99417	C-Myc-binding protein	MYCBP	2	1	1	1	18.2	12.755	0	5.7634
P35580;P35580-3;P35580-5;P35580-2;P35580-4	Myosin-10	MYH10	18	47	37	34	33.6	229	0	323.31
Q7Z406;Q7Z406-6;Q7Z406-2;M0QY43;Q7Z406-	Myosin-14	MYH14	6	7	1	1	3.7	227.87	0	3.1574
P35579;P35579-2	Myosin-9	MYH9	4	72	72	61	43.1	226.53	0	323.31
J3QRS3;P19105;O14950;P24844;J3KTJ1	Myosin regulatory light chain 12A;Myosin regulatory light chain 12B;Myosin regulatory light polypeptide 9	MYL12A;MYL12B;MYL9	6	8	8	8	61	20.457	0	177.11
P60660-2;F8W1R7;G8JLA2;G3V1V0;P60660;J3KND3;B7Z6Z4;G3V1Y7;F8VPF3;F8VZU9;F8W180	Myosin light polypeptide 6	MYL6	15	8	8	8	67.5	16.961	0	132.23
Q92614-3;Q92614-4;Q92614;Q92614-5;Q92614-2	Unconventional myosin-XVIIIa	MYO18A	5	3	3	3	3	226.69	0	5.8351
E9PDF6;O43795;O43795-2	Unconventional myosin-Ib	MYO1B	8	35	35	32	40.1	128.48	0	323.31
F5H6E2;O00159-3;O00159;O00159-2	Unconventional myosin-Ic	MYO1C	10	42	42	41	51.5	118.99	0	323.31
O94832;J3QRN6;K7EIG7	Unconventional myosin-Id	MYO1D	5	54	54	52	59.6	116.2	0	323.31
A0A087WY00;G3V394;F8WE88;F8W6H6;Q9Y4I1-2;Q9Y4I1;Q9Y4I1-3	Unconventional myosin-Va	MYO5A	9	6	5	5	4.4	212.2	0	50.58
Q9ULV0;Q7Z7A5	Unconventional myosin-Vb	MYO5B	6	11	11	10	9.8	213.67	0	33.974
Q9NQX4;Q9NQX4-2	Unconventional myosin-Vc	MYO5C	2	2	1	1	1.1	202.81	0.008532	1.8071
A0A0A0MRM8;Q9UM54-6;E7EW20;Q9UM54-5;Q9UM54-2;Q9UM54-4;Q9UM54-1;Q9UM54	Unconventional myosin-VI	MYO6	9	50	50	50	49.2	145.01	0	323.31
H0YHC3;F8W020;F8W118;F8VXI6;F8VUX1;F8VY35;F8VV59;B7Z9C2;F8W0J6;F5H4R6;H0YIV4;P55209-3;P55209-2;P55209;F8VRJ2;H0YH88;F8W543	Nucleosome assembly protein 1-like 1	NAP1L1	28	3	3	3	22.7	23.417	0	6.9336
A0A087WV29;Q9H0A0-2;Q9H0A0	N-acetyltransferase 10	NAT10	3	1	1	1	3.1	93.533	0	2.5208
P19338;H7BY16	Nucleolin	NCL	2	5	5	5	7.5	76.613	0	8.8735
O00483	Cytochrome c oxidase subunit NDUFA4	NDUFA4	1	2	2	2	27.2	9.3697	0	5.3116
B1AHD1;P55769	NHP2-like protein 1;NHP2-like protein 1, N-terminally	NHP2L1	2	2	2	2	18.2	14.627	0	36.905
Q8N5F7	NF-kappa-B-activating protein	NKAP	1	2	2	2	10.8	47.138	0	4.2577
A0A087WV73;P46087-2;P46087;P46087-	Probable 28S rRNA (cytosine(4447)-C(5))-	NOP2	5	4	4	4	6.3	94.095	0	7.0693
O00567;H0Y653;H0YDU4	Nucleolar protein 56	NOP56	4	8	8	8	20.2	66.049	0	38.383
Q9Y2X3	Nucleolar protein 58	NOP58	2	6	6	6	16.6	59.578	0	16.247
P06748-2;P06748;P06748-3	Nucleophosmin	NPM1	5	5	5	5	28.7	29.464	0	65.973
Q15406-3;Q15406-5;Q15406-2;Q15406-4;Q15406	Nuclear receptor subfamily 6 group A member 1	NR6A1	5	1	1	1	2.9	52.163	0.009449	1.4526

Q7Z417	Nuclear fragile X mental retardation-interacting protein 2	NUFIP2	1	3	3	3	6.6	76.12	0	7.0778
E9PF10;O75694-2;O75694	Nuclear pore complex protein Nup155	NUP155	3	5	5	5	5.9	148.09	0	7.2275
Q8TEM1	Nuclear pore membrane glycoprotein 210	NUP210	1	1	1	1	0.8	205.11	0.009464	1.4607
M0QXN5;P37198	Nuclear pore glycoprotein p62	NUP62	2	1	1	1	5.4	45.615	0	5.0964
H3BVG0;Q8N1F7;Q8N1F7-2;H3BM93	Nuclear pore complex protein Nup93	NUP93	11	4	4	4	7.7	99.554	0	6.1219
F8VUA7;F8VQX7;Q9BZF1-3;Q9BZF1-2;Q9BZF1	Oxysterol-binding protein;Oxysterol-binding protein-related protein 8	OSBPL8	5	1	1	1	2.7	79.052	0	3.0259
A0A087WUD3;Q9NRP0;Q9NRP0-2	Oligosaccharyltransferase complex subunit OSTC	OSTC	3	1	1	1	14.5	9.369	0.003718	2.3002
Q01804-5;Q01804-3;Q01804	OTU domain-containing protein 4	OTUD4	3	2	2	2	3.1	116.95	0.00821	1.6889
P11940;A0A087WTT1;E7EQV3;P11940-	Polyadenylate-binding protein 1;Polyadenylate-binding	PABPC1	27	20	20	13	42.1	70.67	0	323.31
B1ANR0;Q13310-2;Q13310;Q13310-3;H0Y5F5	Polyadenylate-binding protein;Polyadenylate-binding	PABPC4	9	13	6	6	28.1	67.97	0	13.276
Q86U42-2;Q86U42;B4DEH8;G3V4T2;Q92843-2	Polyadenylate-binding protein 2	PABPN1	6	4	4	4	36.5	31.496	0	129
P09874	Poly [ADP-ribose] polymerase 1	PARP1	3	30	30	30	45.1	113.08	0	323.31
B0QYP8		PARVB	1	1	1	1	5.7	21.561	0.008197	1.6793
Q15365	Poly(rC)-binding protein 1	PCBP1	1	4	4	3	15.2	37.497	0	12.363
F8W0G4;F8VXH9;F8W1G6;H3BRU6;F8VZX2;Q15366-7;Q15366-8;Q15366-4;Q15366-5;Q15366-6;Q15366-3;Q15366;Q15366-2;F8VTZ0;J3QT27;E9PFP8;P57721-2;P57721-	Poly(rC)-binding protein 2;Poly(rC)-binding protein 3	PCBP2;PCBP3	21	2	1	1	15.2	16.637	0.008157	1.666
P12004	Proliferating cell nuclear antigen	PCNA	1	1	1	1	4.2	28.768	0.008432	1.7562
Q14690	Protein RRP5 homolog	PDCD11	1	3	3	3	2.2	208.7	0	5.5555
F8WF02;C9J634;P11177-3;P11177-2;P11177	Pyruvate dehydrogenase E1 component subunit beta,	PDHB	5	1	1	1	6.4	27.65	0.008489	1.8016
P30101	Protein disulfide-isomerase A3	PDIA3	2	8	8	8	19.4	56.782	0	12.619
Q15084-3;Q15084;Q15084-4;Q15084-5;Q15084-2	Protein disulfide-isomerase A6	PDIA6	5	1	1	1	3.4	47.837	0.008361	1.738
C9IYB4		PDK1	1	1	1	1	6.6	26.268	0.008278	1.7269
B3KXD6;B5MCF9;O00541-2;O00541	Pescadillo homolog	PES1	4	2	2	2	7.3	51.464	0	2.5717
Q96HS1;Q96HS1-2;F5GXG4	Serine/threonine-protein phosphatase PGAM5,	PGAM5	3	13	13	13	57.8	32.004	0	323.31
O00264;O00264-2	Membrane-associated progesterone receptor component	PGRMC1	2	2	2	2	11.3	21.671	0	3.1027
O15173;O15173-2	Membrane-associated progesterone receptor component	PGRMC2	2	1	1	1	8.5	23.818	0.008091	1.6214
Q7RTV0	PHD finger-like domain-containing protein 5A	PHF5A	1	2	2	2	26.4	12.405	0	45.367
Q8WWQ0	PH-interacting protein	PHIP	1	1	1	1	1	206.69	0.008224	1.6916
E9PJ24;F8WEF5;Q9P1Y6-2;Q9P1Y6-3;Q9P1Y6	PHD and RING finger domain-containing protein 1	PHRF1	5	7	7	7	8.2	178.22	0	13.117
B8QGS9;Q99959-2;Q99959	Plakophilin-2	PKP2	3	2	2	2	2.5	91.239	0	2.6571
Q15149-7;Q15149-8;Q15149-9;Q15149-5;Q15149-4;Q15149-6;Q15149-3;Q15149-2;Q15149	Plectin	PLEC	13	65	65	61	19.2	512.6	0	191.72
D6RA26;O43660	Pleiotropic regulator 1	PLRG1	2	1	1	1	15.6	23.52	0	8.9079
Q9H307;Q9H307-2	Pinin	PNN	4	10	10	10	19.8	81.613	0	93.963
Q8IY17-5;Q8IY17-2;Q8IY17-3;Q8IY17;Q8IY17-4	Neuropathy target esterase	PNPLA6	5	1	1	1	2.8	143.35	0.008834	2.0346
F6VRR5;Q9BY77;Q9BY77-2;F8WCX5;Q8WUT1	Polymerase delta-interacting protein 3	POLDIP3	7	12	12	12	39	48.103	0	119.37
K7EMH3;O00411	DNA-directed RNA polymerase, mitochondrial	POLRMT	2	1	1	1	6.8	39.348	0	12.55
J3QT77;Q15165-3;Q15165-1;Q15165	Serum paraoxonase/arylesterase 2	PON2	4	1	1	1	4.7	37.98	0	2.5326
C9JN15;Q13427-2;Q13427;E9PG73;C9JT64;C9J679;C9JM79	Peptidyl-prolyl cis-trans isomerase;Peptidyl-prolyl cis-trans isomerase G	PPIG	7	4	4	4	20.3	27.409	0	11.202
B8ZZF0;C9JIR6;O75688-5;O75688-4;O75688-	Protein phosphatase 1B	PPM1B	6	1	1	1	4.3	33.232	0.003724	2.3005
F8VR82;F8VYE8;F8W0W8;P36873;P36873-2;A0A087WYY5;E9PMD7;P62140;P62136;P62136-2	Serine/threonine-protein phosphatase;Serine/threonine-protein phosphatase PP1-gamma catalytic subunit;Serine/threonine-protein phosphatase PP1-beta catalytic subunit;Serine/threonine-protein phosphatase	PPP1CC;PPP1CA;PPP1CB	17	3	3	3	13	30.987	0	21.074
D3DTX6;Q96SB3	Neurabin-2	PPP1R9B	2	7	7	7	13.6	89.333	0	18.27
P63151;P63151-2;E5RIY1;F5GXJ6;E5RFR9;Q00005-6;Q00005;Q00005-2;Q00005-3;Q66LE6;Q00005-4;Q00005-5;Q00005-7	Serine/threonine-protein phosphatase 2A 55 kDa regulatory subunit B alpha isoform;Serine/threonine-protein phosphatase 2A 55 kDa regulatory subunit B beta isoform;Serine/threonine-protein phosphatase 2A 55 kDa	PPP2R2A;PPP2R2B;PPP2R2D	13	2	2	2	5.6	51.691	0	6.1027
Q06830;A0A0A0MSI0;A0A0A0MRQ5	Peroxiredoxin-1	PRDX1	4	4	4	4	27.1	22.11	0	7.9714
Q9HCU5;B5MC98	Prolactin regulatory element-binding protein	PREB	4	4	4	4	16.3	45.468	0	17.868
H3BV73;P05771;P17252;P05771-2	Protein kinase C beta type;Protein kinase C alpha type	PRKCB;PRKCA	4	1	1	1	12.3	14.888	0	2.4347
P78527-2;P78527	DNA-dependent protein kinase catalytic subunit	PRKDC	2	13	13	13	5.2	465.5	0	30.707
E9PKG1;H7C2I1;Q99873-3;Q99873-2;Q99873-4;Q99873;E9PIX6;E9PQ98;A0A087X1W2	Protein arginine N-methyltransferase 1	PRMT1	13	9	9	9	34.8	37.709	0	24.166
O14744;O14744-5;O14744-2;O14744-3;G3V580;H0YJX6	Protein arginine N-methyltransferase 5;Protein arginine N-methyltransferase 5, N-terminally processed	PRMT5	15	7	7	7	16.2	72.683	0	25.349
Q9UMS4	Pre-mRNA-processing factor 19	PRPF19	5	15	15	15	63.7	55.18	0	323.31
E7EU94;E7ESX0;E7EN72;E7EVX8;Q8WWY3-3;Q8WWY3-2;Q8WWY3-4;Q8WWY3	U4/U6 small nuclear ribonucleoprotein Prp31	PRPF31	8	1	1	1	8.1	28.858	0	4.6441
Q8NAV1	Pre-mRNA-splicing factor 38A	PRPF38A	1	3	3	3	11.5	37.476	0	6.3509
Q5VTL8;Q5VTL8-2	Pre-mRNA-splicing factor 38B	PRPF38B	3	3	3	3	11.5	64.467	0	7.4191
Q13523;H0YDJ3	Serine/threonine-protein kinase PRP4 homolog	PRPF4B	2	6	6	6	7	116.99	0	10.309
O94906-2;O94906	Pre-mRNA-processing factor 6	PRPF6	2	3	3	3	4.1	102.43	0	5.4059
Q6P2Q9	Pre-mRNA-processing-splicing factor 8	PRPF8	4	25	25	25	16.1	273.6	0	147.71
Q6MZQ0-2;Q6MZQ0-3;Q6MZQ0	Proline-rich protein 5-like	PRR5L	3	1	1	1	14.4	15.064	0.00817	1.6695
P48634-3;P48634;P48634-4;P48634-2	Protein PRRC2A	PRRC2A	4	7	7	7	6.2	229.06	0	11.988
H7C5N8;E7EPN9;Q9Y520-2;Q9Y520-3;Q9Y520-6;Q9Y520-4;Q9Y520-5;Q9Y520;Q9Y520-7	Protein PRRC2C	PRRC2C	9	1	1	1	1.5	135.86	0	4.5345
H0YMZ1;H0YN18;H0YL69;P25789;H0YLS6;H0YLC2;H0YKT8;H0YMI6;H0YMA1;P25789-2	Proteasome subunit alpha type;Proteasome subunit alpha type-4;Proteasome subunit beta type	PSMA4	10	2	2	2	8.2	24.526	0	2.4093
P43686-2;P43686	26S protease regulatory subunit 6B	PSMC4	2	1	1	1	4.7	43.507	0.008787	2.0128
F8WBS8;C9JPC0;Q13200	26S proteasome non-ATPase regulatory subunit 2	PSMD2	3	1	1	1	39	6.5831	0	2.7063
O43242-2;O43242	26S proteasome non-ATPase regulatory subunit 3	PSMD3	2	2	2	2	6.7	41.183	0	6.3912
A6NLN1;P26599;P26599-2;P26599-3;K7EKJ7	Polypyrimidine tract-binding protein 1	PTBP1	16	11	11	11	38.7	56.51	0	171.93
P48651-3;P48651-2;P48651	Phosphatidylserine synthase 1	PTDSS1	3	2	2	2	9	34.579	0	4.0089
B4DSN5;P18031	Tyrosine-protein phosphatase non-receptor type;Tyrosine-protein phosphatase non-receptor type 1	PTPN1	2	2	2	2	6.6	41.26	0	4.3239
Q5W0J0;A0A087X163;Q9NP72-	Ras-related protein Rab-18	RAB18	6	2	2	2	18.6	18.023	0	24.187
P51148;P51148-2;K7ENY4;K7ERI8;K7ERQ8	Ras-related protein Rab-5C	RAB5C	13	5	5	5	36.6	23.482	0	42.267
C9JB90;C9JU14;J3KR73;H7BYW1;H0YGL6;P20340-4;Q9NRW1-2;Q9NRW1;P20340-2;P20340;Q14964	Ras-related protein Rab-6A;Ras-related protein Rab-6B;Ras-related protein Rab-39A	RAB6B;RAB6A;RAB39A	11	1	1	1	22.4	5.8575	0	8.2355
Q9P0K7-4;Q9P0K7-3;Q9P0K7;Q9P0K7-2	Ankycorbin	RAI14	4	6	6	6	9.5	106.9	0	15.523
Q15311	RalA-binding protein 1	RALBP1	1	1	1	1	3.8	76.063	0	2.4113
Q5QPM2;Q5QPM1;Q5QPL9;Q9UKM9-2;Q9UKM9	RNA-binding protein Raly	RALY	5	1	1	1	12.4	9.714	0.008104	1.6243
B5MDF5;P62826;J3KQE5;F5H018	GTP-binding nuclear protein Ran	RAN	6	5	5	5	24	26.224	0	8.9239
P49792	E3 SUMO-protein ligase RanBP2	RANBP2	10	11	11	8	5.6	358.2	0	26.708
P46060	Ran GTPase-activating protein 1	RANGAP1	1	3	3	3	7.5	63.541	0	5.6254
F5H823;B7ZB78;F5GZG1;P61224-2;P61224-4;P61224-3;P62834;P61224;A6NIZ1	Ras-related protein Rap-1b;Ras-related protein Rap-1A;Ras-related protein Rap-1b-like protein	RAP1B;RAP1A	9	1	1	1	11.7	11.916	0.008418	1.7505
A0A0A0MR66;P98175-2;P98175;P98175-	RNA-binding protein 10	RBM10	5	20	20	19	27.8	110.36	0	323.31
Q96PK6	RNA-binding protein 14	RBM14	8	8	8	7	20.2	69.491	0	24.558
Q96PK6-5;Q9BWF3	RNA-binding protein 14;RNA-binding protein 4	RBM14;RBM4	2	2	1	1	9.4	37.034	0	4.508
A0A087WWP4;Q96T37-4;Q96T37-2;Q96T37-	Putative RNA-binding protein 15	RBM15	5	4	4	4	7.7	102.13	0	7.4787
Q96I25;Q5W011;Q5W010	Splicing factor 45	RBM17	5	9	9	9	27.7	44.961	0	27.538
P49756;E9PQU5;P49756-2;P49756-3	RNA-binding protein 25	RBM25	4	3	3	3	5.9	100.18	0	5.393
Q9NW13-2;Q9NW13	RNA-binding protein 28	RBM28	2	2	2	2	3.4	69.922	0	2.5274
Q14498-3;Q14498-	RNA-binding protein 39	RBM39	7	6	6	6	21.7	57.089	0	87.65
Q9Y5S9-2;Q9Y5S9	RNA-binding protein 8A	RBM8A	2	4	4	4	46.2	19.76	0	35.3
H3BR27;H0Y6E7;H3BT71;P38159-3;P38159;P38159-2;O75526	RNA-binding motif protein, X chromosome;RNA-binding motif protein, X chromosome, N-terminally processed;RNA-binding motif protein, X-linked-like-2	RBMX;RBMXL2	10	3	3	3	38.5	8.6098	0	26.493
Q14257;Q14257-2;H0YL43;A8MXP8	Reticulocalbin-2	RCN2	4	6	6	6	27.4	36.876	0	66.013
Q8TC12;G3V2G6;Q8TC12-3;Q8TC12-2	Retinol dehydrogenase 11	RDH11	6	3	3	3	14.8	35.386	0	5.8122
Q5T092;A0A0A0MR06;Q5T091;Q9P0H9;O15258;	Protein RER1	RER1	6	2	2	2	23.4	18.388	0	6.2068
J3KNE0;A6NKT7;Q7Z3J3	RanBP2-like and GRIP domain-containing protein 3;RanBP2-like and GRIP domain-containing protein 4	RGPD3;RGPD4	3	4	1	1	3.1	197.62	0.009375	1.4351
H7BZ48		RGPD6	1	1	1	1	9.9	21.726	0.008389	1.749
Q5UIP0-2;Q5UIP0;H7C2B5	Telomere-associated protein RIF1	RIF1	4	5	5	5	4	271.69	0	6.5792
Q99942	E3 ubiquitin-protein ligase RNF5	RNF5	1	1	1	1	8.3	19.881	0	3.5984
H3BV80;H3BMM9;H3BTC0;Q15287-3;Q15287-2;Q15287;H3BPG5;H3BMS0	RNA-binding protein with serine-rich domain 1	RNPS1	8	5	5	5	33.6	24.561	0	119.84
X1WI28;P27635;B8A6G2;F8W7C6;A0A087WV22;	60S ribosomal protein L10	RPL10	9	4	4	4	26.5	22.975	0	94.28
P62913-2;P62913;Q5VVC9;Q5VVC8	60S ribosomal protein L11	RPL11	4	4	4	4	20.9	20.124	0	29.947
P30050;P30050-2	60S ribosomal protein L12	RPL12	2	5	5	5	44.8	17.818	0	25.71
P26373;J3QSB4;P26373-2	60S ribosomal protein L13	RPL13	4	4	4	4	19	24.261	0	28.662
Q8J015;M0QYS1;Q6NVV1;P40429	Putative 60S ribosomal protein L13a protein RPL13AP3;60S ribosomal protein L13a	RPL13a;RPL13A;RPL13AP3	4	2	2	2	15.5	16.731	0	4.8561
E7EPB3;P50914	60S ribosomal protein L14	RPL14	2	2	2	2	18.5	14.558	0	4.9637
E7EQV9;E7ENU7;P61313;E7EX53	Ribosomal protein L15;60S ribosomal protein L15	RPL15	4	2	2	2	14.9	20.51	0	5.9107
A0A087WXM6;J3QQT2;J3KRX5;A0A0A6YYL6;P18621;P18621-3;J3KRB3;A0A087WWH0;J3QS96;J3QLC8;A0A0A0	60S ribosomal protein L17	RPL17;RPL17-C18orf32	14	5	5	5	34.3	19.586	0	107.99
G3V203;H0YHA7;J3QQ67;Q07020-2;Q07020;F8VUA6;F8VYV2;A0A075B7A0	60S ribosomal protein L18	RPL18	8	4	4	4	29.3	18.756	0	56.46
J3QL15;J3QR09;J3KTE4;P84098	Ribosomal protein L19;60S ribosomal protein L19	RPL19	4	1	1	1	13.3	15.003	0	102.55
G3V1B3;M0R181;P46778	60S ribosomal protein L21	RPL21	3	1	1	1	17.2	9.8864	0	44.514
K7EJT5;K7EP65;K7EKS7;K7ELC4;K7EMH1;K7ERI7;P	60S ribosomal protein L22	RPL22	7	2	2	2	51.1	5.0827	0	4.5266

P62829;C9JD32;B9ZVP7;J3KT29	60S ribosomal protein L23	RPL23	5	4	4	4	36.4	14.865	0	98.077
K7EMA7;H7BY10;K7EJV9;K7ERT8;A8MUS3;P6275	60S ribosomal protein L23a	RPL23A	6	4	4	4	51.4	7.9232	0	8.1733
C9JXB8;C9JNW5;P83731	60S ribosomal protein L24	RPL24	3	4	4	4	33.1	14.369	0	10.178
P61353;K7ELC7;K7EQQ9	60S ribosomal protein L27	RPL27	4	4	4	4	36	15.798	0	20.071
E9PJD9;E9PLL6;P46776	60S ribosomal protein L27a	RPL27A	4	3	3	3	36.3	10.127	0	39.663
H0YLP6;H0YMF4;H0YKD8;P46779-4;P46779-5;P46779;P46779-2;P46779-3	60S ribosomal protein L28	RPL28	8	1	1	1	11.2	9.657	0.008818	2.0283
P47914	60S ribosomal protein L29	RPL29	1	1	1	1	9.4	17.752	0	7.5483
P39023;H7C422;H7C3M2;G5E9G0;F8WCR1;B5MC	60S ribosomal protein L3	RPL3	7	7	7	7	24.1	46.108	0	26.451
A0A0B4J213;E5RI99;P62888	60S ribosomal protein L30	RPL30	3	1	1	1	37.3	5.5553	0	5.5656
H7C2W9;C9JU56;B7Z4E3;B7Z4C8;P62899-	60S ribosomal protein L31	RPL31	7	1	1	1	13	12.701	0	3.1456
D3YTB1;F8W727;P62910	60S ribosomal protein L32	RPL32	3	3	3	3	27.8	15.616	0	6.1583
F2Z388;P42766	60S ribosomal protein L35	RPL35	2	2	2	2	24	10.645	0	2.3868
J3QSB5;Q9Y3U8;J3KTD3	60S ribosomal protein L36	RPL36	3	2	2	2	20.2	10.789	0.00713	2.1273
C9J4Z3;P61513	60S ribosomal protein L37a	RPL37A	2	1	1	1	26.5	7.624	0	46.581
P63173;J3KT73;J3QL01	60S ribosomal protein L38	RPL38	4	5	5	5	50	8.2178	0	14.346
Q59GN2;P62891	Putative 60S ribosomal protein L39-like 5;60S ribosomal	RPL39P5;RPL39	2	1	1	1	19.6	6.3225	0	2.3574
P36578;H3BM89	60S ribosomal protein L4	RPL4	4	7	7	7	20.6	47.697	0	14.34
Q02878	60S ribosomal protein L6	RPL6	4	5	5	5	24.7	32.728	0	35.317
A8MUD9;P18124;C9JZ88;C9JIJ5	60S ribosomal protein L7	RPL7	4	4	4	4	23.1	24.433	0	8.7243
P62424;Q5T8U3	60S ribosomal protein L7a	RPL7A	3	5	5	5	20.7	29.995	0	14.577
G3V1A1;E9PKZ0;P62917;E9PKU4	60S ribosomal protein L8	RPL8	4	2	2	2	21.6	18.169	0	4.6506
P05388;F8VZS0;F8VU65;F8VWS0;P05388-2;Q8NHW5;F8VS58;F8VQY6;F8VPE8;F8VRK7;G3V	60S acidic ribosomal protein P0;60S acidic ribosomal protein P0-like	RPLP0;RPLP0P6	14	4	4	4	16.4	34.273	0	13.414
P05387	60S acidic ribosomal protein P2	RPLP2	1	5	5	3	69.6	11.665	0	15.31
P04843;B7Z4L4	Dolichyl-diphosphooligosaccharide--protein glycosyltransferase subunit 1	RPN1	3	13	13	13	35.4	68.569	0	51.592
P04844-2;P04844	Dolichyl-diphosphooligosaccharide--protein glycosyltransferase subunit 2	RPN2	5	8	8	8	22	67.723	0	22.782
P46783;F6U211;S4R435	40S ribosomal protein S10	RPS10;RPS10-NUDT3	4	3	3	3	23.6	18.898	0	7.2305
P62280;M0QZC5;M0R1H5	40S ribosomal protein S11	RPS11	3	6	6	6	36.1	18.431	0	16.437
P25398	40S ribosomal protein S12	RPS12	1	1	1	1	11.4	14.515	0.008	1.5243
P62277;J3KMX5	40S ribosomal protein S13	RPS13	3	7	7	7	42.4	17.222	0	16.963
P62263;E5RH77	40S ribosomal protein S14	RPS14	3	7	7	7	58.9	16.273	0	102.36
K7EM56;A0A0B4J2B4;S4R417;K7ELC2;P62841;S4R456;K7EJ78;K7EQJ5	40S ribosomal protein S15	RPS15	8	2	2	2	34.8	12.99	0	3.8727
P62244;I3L3P7;I3L246;H3BN98	40S ribosomal protein S15a	RPS15A	8	5	5	5	46.2	14.839	0	29.853
P62249;M0R210;M0R3H0;A0A087WZ27;M0R1M5	40S ribosomal protein S16	RPS16;ZNF90	7	9	9	9	54.1	16.445	0	34.075
H0YN88;A0A075B716;P0CW22;P08708;H0YN73	40S ribosomal protein S17-like;40S ribosomal protein S17	RPS17;RPS17L	5	4	4	4	33.1	15.919	0	8.1324
P62269	40S ribosomal protein S18	RPS18	2	5	5	5	32.2	17.718	0	105.51
P39019;M0R2L9;M0QXK4;M0QYF7;M0R140	40S ribosomal protein S19	RPS19	6	3	3	3	21.4	16.06	0	7.372
P15880;H0YEN5;E9PQD7;I3L404;E9PMM9	40S ribosomal protein S2	RPS2	9	11	11	11	39.9	31.324	0	316.52
P60866;P60866-2;E5RIP1;E5RJX2	40S ribosomal protein S20	RPS20	5	3	3	3	22.7	13.373	0	21.901
P62266;D6RD47	40S ribosomal protein S23	RPS23	2	3	3	3	27.3	15.807	0	87.786
E7ETK0;A0A087WUS0;P62847-2;P62847-	40S ribosomal protein S24	RPS24	6	2	2	2	20.6	15.197	0	7.2701
P62851	40S ribosomal protein S25	RPS25	1	4	4	4	24	13.742	0	37.881
Q5T4L4;P42677	40S ribosomal protein S27	RPS27	2	3	3	2	37.9	7.3564	0	25.464
P62979;J3QS39;J3QTR3;F5H6Q2;F5GYU3;F5H2Z3;F5H265;B4DV12;F5H388;F5H747;F5GXK7;J3QKN0;Q5PY61;Q96C32;P62987;P0CG47;P0CG48;M0R1V7;A0A087WV77;M0R1M6;M0R2S1;J3QLP7;J3QR	Ubiquitin-40S ribosomal protein S27a;Ubiquitin;40S ribosomal protein S27a;Ubiquitin-60S ribosomal protein L40;Ubiquitin;60S ribosomal protein L40;Polyubiquitin-B;Ubiquitin;Polyubiquitin-C;Ubiquitin	RPS27A;UBB;UBC;UBA52;UBBP4	27	4	4	4	30.1	17.965	0	95.008
C9JLI6;H0YMV8;Q71UM5	40S ribosomal protein S27;40S ribosomal protein S27-like	RPS27L	3	2	1	1	24.7	10.886	0	15.404
P62857	40S ribosomal protein S28	RPS28	1	1	1	1	17.4	7.8409	0.009479	1.4623
P23396;P23396-2;E9PL09;E9PPU1;H0YEU2;F2Z2S8;H0YCJ7;H0YF3	40S ribosomal protein S3	RPS3	14	12	12	12	60.5	26.688	0	123.78
D6RB09;D6RG13;D6RAT0;P61247;D6RED7;D6RAS7;H0Y8L7;E9PFI5	40S ribosomal protein S3a	RPS3A	12	3	3	3	22.8	22.459	0	20.726
P62701	40S ribosomal protein S4, X isoform	RPS4X	5	8	8	8	35.7	29.597	0	61.545
M0QZN2;M0R0F0;M0R0R2;P46782	40S ribosomal protein S5;40S ribosomal protein S5, N-terminally processed	RPS5	4	4	4	4	40.3	14.763	0	30.838
P62753;A2A3R5;A2A3R7	40S ribosomal protein S6	RPS6	3	4	4	4	22.5	28.68	0	63.065
P62081;B5MCP9	40S ribosomal protein S7	RPS7	2	4	4	4	27.8	22.127	0	14.371
P62241;Q5JR95	40S ribosomal protein S8	RPS8	2	7	7	7	41.3	24.205	0	182.39
P46781;B5MCT8;C9JM19	40S ribosomal protein S9	RPS9	5	7	7	7	28.4	22.591	0	12.79
P56182	Ribosomal RNA processing protein 1 homolog A	RRP1	1	1	1	1	2.8	52.839	0.008803	2.0199
Q14684-2;Q14684	Ribosomal RNA processing protein 1 homolog B	RRP1B	2	3	3	3	5.5	82.175	0	4.7692
E9PPY3;O43159	Ribosomal RNA-processing protein 8	RRP8	2	1	1	1	6.2	34.2	0.005485	2.2315
Q15050	Ribosome biogenesis regulatory protein homolog	RRS1	1	2	2	2	8.2	41.193	0	13.806
J3QSV6;O76021;I3L234;I3L3C4;I3L3U9	Ribosomal L1 domain-containing protein 1	RSL1D1	6	5	5	5	15.1	48.208	0	14.009
Q9Y3I0	tRNA-splicing ligase RtcB homolog	RTCB	1	3	3	3	7.7	55.21	0	4.1678
P05109	Protein S100-A8;Protein S100-A8, N-terminally processed	S100A8	1	1	1	1	11.8	10.834	0.00726	2.21
Q15424-2;Q15424;Q15424-4;Q15424-3	Scaffold attachment factor B1	SAFB	6	5	5	3	9.9	95.18	0	17.678
Q14151	Scaffold attachment factor B2	SAFB2	3	4	2	2	7.9	107.47	0	3.7641
X6RAL5;O00422;U3KPY7;H7BZW6	Histone deacetylase complex subunit SAP18	SAP18	5	4	4	4	28.5	19.526	0	8.188
O43290;E9PQI8	U4/U6.U5 tri-snRNP-associated protein 1	SART1	2	4	4	4	8.4	90.254	0	5.419
Q9H7N4	Splicing factor, arginine/serine-rich 19	SCAF1	1	2	2	2	3.2	139.27	0	10.221
Q9Y6U3;Q9Y6U3-2;Q9Y6U3-3	Adseverin	SCIN	7	14	14	14	28.1	80.488	0	66.039
Q9HCN8	Stromal cell-derived factor 2-like protein 1	SDF2L1	1	1	1	1	10	23.598	0	2.4414
A0A087X1A9;O75396	Vesicle-trafficking protein SEC22b	SEC22B	2	1	1	1	15.2	8.7819	0.008446	1.7821
S4R3B5;P60468	Protein transport protein Sec61 subunit beta	SEC61B	2	1	1	1	23.8	4.8738	0.008666	1.9251
Q9UGP8	Translocation protein SEC63 homolog	SEC63	1	3	3	3	4.1	87.996	0	2.4355
F8WAX7;F6XHT0;Q9Y6D0	Selenoprotein K	SELK	3	1	1	1	16.7	8.2912	0	2.4456
Q15459;Q15459-2	Splicing factor 3A subunit 1	SF3A1	3	7	7	7	14.2	88.885	0	18.269
K7EMT0;Q15428	Splicing factor 3A subunit 2	SF3A2	2	1	1	1	14.1	15.224	0	3.0753
Q12874	Splicing factor 3A subunit 3	SF3A3	1	4	4	4	12.8	58.848	0	15.418
O75533	Splicing factor 3B subunit 1	SF3B1	4	41	41	41	48.2	145.83	0	323.31
E9PPJ0;Q13435;A0A087WZZ5	Splicing factor 3B subunit 2	SF3B2	8	17	17	17	28	98.169	0	286.81
Q15393	Splicing factor 3B subunit 3	SF3B3	8	36	36	36	40.6	135.58	0	323.31
Q15427	Splicing factor 3B subunit 4	SF3B4	2	3	3	3	14.4	44.385	0	49.826
Q9BWJ5	Splicing factor 3B subunit 5	SF3B5	1	2	2	2	32.6	10.135	0	13.405
Q9Y3B4	Splicing factor 3B subunit 6	SF3B6	1	4	4	4	40.8	14.585	0	8.8637
E5RJR5;P63208	S-phase kinase-associated protein 1	SKP1	2	1	1	1	7.4	18.72	0	5.1447
P53985;Q5T8R3;Q5T8R5;P53985-2	Monocarboxylate transporter 1	SLC16A1	4	2	2	2	6.4	53.944	0	5.0927
P53007;B4DP62	Tricarboxylate transport protein, mitochondrial	SLC25A1	2	3	3	3	11.3	34.012	0	4.8094
B4DLN1;Q9UBX3;Q9UBX3-2	Mitochondrial dicarboxylate carrier	SLC25A10	3	2	2	2	7.2	48.099	0	3.1884
I3L1P8;Q02978-2;Q02978	Mitochondrial 2-oxoglutarate/malate carrier protein	SLC25A11	3	1	1	1	5.4	32.182	0	24.591
Q9UJS0;Q9UJS0-2	Calcium-binding mitochondrial carrier protein Aralar2	SLC25A13	5	3	3	3	5.6	74.175	0	3.6573
F8VVM2;Q00325-2;Q00325	Phosphate carrier protein, mitochondrial	SLC25A3	6	7	7	7	29.6	36.161	0	69.735
P05141	ADP/ATP translocase 2;ADP/ATP translocase 2, N-terminally processed	SLC25A5	1	11	6	6	35.2	32.852	0	16.615
P12236	ADP/ATP translocase 3;ADP/ATP translocase 3, N-terminally processed	SLC25A6	1	14	14	5	56	32.866	0	111.65
Q6P1M0;Q6P1M0-2	Long-chain fatty acid transport protein 4	SLC27A4	2	2	2	2	3	72.063	0.008757	1.9959
Q96K37	Solute carrier family 35 member E1	SLC35E1	1	1	1	1	5.9	44.772	0.005535	2.2879
F5GZI0;F5GZS6;J3KPF3;P08195-2;P08195-	4F2 cell-surface antigen heavy chain	SLC3A2	7	1	1	1	12	18.95	0.008503	1.802
H0YLW7;H0YMW8;H0YNF3;H0YMR6;H0YLE6;H0YL55;H7BXE3;Q9NWH9	SAFB-like transcription modulator	SLTM	8	1	1	1	30.6	6.6127	0	22.705
O60264	SWI/SNF-related matrix-associated actin-dependent regulator of chromatin subfamily A member 5	SMARCA5	1	2	2	2	2.6	121.9	0	3.5053
Q8WVI0	Small integral membrane protein 4	SMIM4	1	1	1	1	15.7	8.6961	0.008403	1.7501
E7EQZ4;Q16637-4;Q16637-2;Q16637-	Survival motor neuron protein	SMN1;SMN2	6	2	2	2	10.5	31.689	0	5.5414
Q2TAY7-2;Q2TAY7	WD40 repeat-containing protein SMU1;WD40 repeat-containing protein SMU1, N-terminally processed	SMU1	2	6	6	6	29.5	39.343	0	17.857
O75643	U5 small nuclear ribonucleoprotein 200 kDa helicase	SNRNP200	3	26	26	26	17.9	244.5	0	101.24
Q96DI7;Q96DI7-2;Q9NSS8	U5 small nuclear ribonucleoprotein 40 kDa protein	SNRNP40;DKFZp434D199	3	6	6	6	30.8	39.31	0	38.218
P08621-2;P08621;P08621-3;M0QYR1	U1 small nuclear ribonucleoprotein 70 kDa	SNRNP70	5	4	4	4	13.1	50.617	0	9.9562
P09661;H0YMA0;H0YLR3;H0YKK0	U2 small nuclear ribonucleoprotein A	SNRPA1	4	6	6	6	33.7	28.415	0	7.7091
P08579	U2 small nuclear ribonucleoprotein B	SNRPB2	1	1	1	1	8.4	25.486	0	3.0669
P62314;J3QLI9	Small nuclear ribonucleoprotein Sm D1	SNRPD1	3	4	4	4	54.6	13.281	0	38.857
P62316;K7ERG4;P62316-2	Small nuclear ribonucleoprotein Sm D2	SNRPD2	4	3	3	3	32.2	13.527	0	4.7448
P62318-2;P62318	Small nuclear ribonucleoprotein Sm D3	SNRPD3	3	3	3	3	33.3	13.291	0	44.636
P62304;A6NHK2	Small nuclear ribonucleoprotein E	SNRPE	2	2	2	2	25	10.803	0	2.9749
P62306	Small nuclear ribonucleoprotein F	SNRPF	1	1	1	1	15.1	9.7251	0	15.16
J3QLE5;A8MT02;P14678-2;P63162;P14678;P63162-2;P14678-	Small nuclear ribonucleoprotein-associated proteins B and B;Small nuclear ribonucleoprotein-associated protein	SNRPN;SNRPB	9	4	4	4	26	17.546	0	4.515
G3V3A4;Q13573;G3V4X8	SNW domain-containing protein 1	SNW1	4	4	4	4	10.3	65.391	0	60.052
Q5QPE4;Q5QPE5;Q9Y5X3	Sorting nexin-5	SNX5	3	1	1	1	4.8	26.596	0.008013	1.5367
P18583-8;P18583-6;P18583-2;P18583-10;P18583-3;P18583-4;P18583-7;P18583;P18583-5;P18583-9	Protein SON	SON	10	2	2	2	4.4	73.883	0	3.1048
Q13813-3;Q13813;Q13813-2	Spectrin alpha chain, non-erythrocytic 1	SPTAN1	3	17	17	17	10.3	282.28	0	40.283
A0A087WUZ3;Q01082;Q01082-3;Q01082-2	Spectrin beta chain, non-erythrocytic 1	SPTBN1	5	10	10	10	6.5	274.83	0	16.694

O15269	Serine palmitoyltransferase 1	SPTLC1	2	3	3	3	11.6	52.743	0	8.1338
P08240-2;P08240	Signal recognition particle receptor subunit alpha	SRPR	2	3	3	3	6.1	66.558	0	4.4111
Q9Y5M8;H7C4H2	Signal recognition particle receptor subunit beta	SRPRB	3	8	8	8	38.7	29.702	0	40.615
M0R088;E9PCT1;A9Z1X7;Q8IYB3-	Serine/arginine repetitive matrix protein 1	SRRM1	7	2	2	2	4.3	78.139	0	7.0922
Q9UQ35;Q9UQ35-2	Serine/arginine repetitive matrix protein 2	SRRM2	9	21	21	21	11.6	299.61	0	297.51
Q9BXP5-5;Q9BXP5-4;Q9BXP5-2;Q9BXP5-	Serrate RNA effector molecule homolog	SRRT	5	1	1	1	2.3	96.221	0.008518	1.807
J3KTL2;Q07955-3;Q07955;Q07955-2	Serine/arginine-rich splicing factor 1	SRSF1	7	8	8	8	36	28.329	0	24.525
Q5JRI1;O75494-5;O75494-4;O75494-6;O75494-3;O75494-2;O75494	Serine/arginine-rich splicing factor 10	SRSF10	10	6	6	6	36	20.913	0	37.875
J3QL05;J3KP15;Q01130-2;Q01130	Serine/arginine-rich splicing factor 2	SRSF2	6	3	3	3	31.5	15.156	0	12.515
A0A087X2D0;P84103-2;P84103	Serine/arginine-rich splicing factor 3	SRSF3	3	4	4	3	49.5	10.32	0	65.717
Q13247-3;Q13247	Serine/arginine-rich splicing factor 6	SRSF6	8	5	5	5	14.9	38.418	0	16.551
A0A0B4J1Z1;C9JAB2;Q16629-3;Q16629-2;Q16629-	Serine/arginine-rich splicing factor 7	SRSF7	6	4	3	3	28.5	15.763	0	6.7033
Q13242;H0YIB4;S4R3G0	Serine/arginine-rich splicing factor 9	SRSF9	3	4	4	4	22.2	25.542	0	8.5275
P51571	Translocon-associated protein subunit delta	SSR4	1	1	1	1	7.5	18.998	0.008726	1.9808
A0A087X1A5;O95793-2;O95793-3;O95793;F6UDC6;Q5JW28;Q5JW30	Double-stranded RNA-binding protein Staufen homolog 1	STAU1	7	2	2	2	6.5	54.945	0	2.4762
Q15208	Serine/threonine-protein kinase 38	STK38	3	14	14	12	43.9	54.19	0	256.38
Q9Y2H1-2;Q9Y2H1	Serine/threonine-protein kinase 38-like	STK38L	2	3	1	1	7.8	43.17	0.007156	2.1424
F8VSL7;P27105	Erythrocyte band 7 integral membrane protein	STOM	2	2	2	2	19.6	18.699	0	6.9118
Q8TCJ2	Dolichyl-diphosphooligosaccharide--protein glycosyltransferase subunit STT3B	STT3B	1	1	1	1	1.5	93.673	0	5.3701
Q9UNE7;Q9UNE7-2;H3BS86	E3 ubiquitin-protein ligase CHIP	STUB1	5	5	5	5	20.1	34.856	0	7.9767
P53999	Activated RNA polymerase II transcriptional coactivator	SUB1	1	2	2	2	18.9	14.395	0	5.3843
Q9Y5B9	FACT complex subunit SPT16	SUPT16H	1	2	2	2	2.8	119.91	0	2.4885
O95425-2;O95425-4;O95425-3;O95425	Supervillin	SVIL	4	28	28	28	24.6	200.84	0	323.31
B7Z645;O60506-4;O60506-3;O60506-	Heterogeneous nuclear ribonucleoprotein Q	SYNCRIP	7	6	4	4	19.8	52.046	0	16.302
E7ENN3;A0A0C4DG40;Q8NF91-4;Q8NF91;Q8NF91-8;Q8NF91-2;Q8NF91-7	Nesprin-1	SYNE1	7	2	2	2	0.2	964.83	0.008052	1.5888
A0A075B7D9;H3BPE7;P35637-2;P35637;Q92804-	RNA-binding protein FUS;TATA-binding protein-	TAF15;FUS	6	2	2	2	5.1	48.839	0	4.6142
A0A087WX29;Q13148-4;Q13148	TAR DNA-binding protein 43	TARDBP	3	1	1	1	7.4	26.743	0	3.8
E9PF19;Q9Y4P3;Q96E41	Transducin beta-like protein 2	TBL2	4	4	4	4	14.8	45.935	0	10.609
J3KNP2;Q12788;A0A087WYP7	Transducin beta-like protein 3	TBL3	3	3	3	3	5.7	76.979	0	24.622
J3KQ96;E7ETY2;Q13428-2;Q13428-8;Q13428-6;Q13428-7;Q13428;Q13428-3;Q13428-4	Treacle protein	TCOF1	11	5	5	5	6.3	144.12	0	8.9549
Q9NZ01;M0R3C3;M0QXM3;Q9NZ01-2	Very-long-chain enoyl-CoA reductase	TECR	4	2	2	2	7.1	36.034	0	16.217
J3KNF3;O43151-2;O43151;O43151-3	Methylcytosine dioxygenase TET3	TET3	4	2	2	2	1.6	183.58	0.008237	1.7018
C9JL43;C9JUC3;C9J7N0;C9JXQ7;C9JHH5;A0A087	Testis-expressed sequence 264 protein	TEX264	7	1	1	1	10.6	11.032	0	2.7079
P02786;G3V0E5	Transferrin receptor protein 1;Transferrin receptor protein 1, serum form	TFRC	3	3	3	3	5.8	84.87	0	4.3493
A0A087WWS1;Q96FV9;R4GMN4	THO complex subunit 1	THOC1	3	2	2	2	6.1	75.651	0	8.4376
Q8NI27;A0A0C4DG98	THO complex subunit 2	THOC2	4	7	7	7	5.3	182.77	0	10.322
Q13769	THO complex subunit 5 homolog	THOC5	1	2	2	2	5.1	78.507	0	6.3802
Q86W42-3;Q86W42-2;Q86W42	THO complex subunit 6 homolog	THOC6	3	2	2	2	10.1	32.89	0	2.6622
Q6I9Y2;F8WF22;A0A024R341	THO complex subunit 7 homolog	THOC7;NIF3L1BP1	3	2	2	2	10.3	23.743	0.007181	2.1592
Q9Y2W1	Thyroid hormone receptor-associated protein 3	THRAP3	1	24	23	23	29.4	108.66	0	323.31
B4DDK6;O14925;Q5SRD1	Mitochondrial import inner membrane translocase subunit Tim23;Putative mitochondrial import inner membrane translocase subunit Tim23B	TIMM23B;TIMM23	3	1	1	1	12.1	14.6	0.00722	2.1715
M0R0C3;M0R2F8;Q3ZCQ8-3;Q3ZCQ8;Q3ZCQ8-2	Mitochondrial import inner membrane translocase	TIMM50	6	3	3	3	30.8	11.866	0	3.2446
A0A087X0K9;G5E9E7;G3V1L9;Q07157-	Tight junction protein ZO-1	TJP1	6	2	2	2	2.3	187.85	0	2.4549
J3QQY2;J9JIE6;Q9UM00-2;Q9UM00;J3KS45	Transmembrane and coiled-coil domain-containing	TMCO1	5	2	2	2	27.9	11.372	0	4.8775
Q9BVK6	Transmembrane emp24 domain-containing protein 9	TMED9	1	1	1	1	4.7	27.277	0	2.6359
D6RAA6;P57088	Transmembrane protein 33	TMEM33	2	1	1	1	5.4	25.223	0	3.0057
Q9NYL9;H0YNJ8;H0YKU1	Tropomodulin-3	TMOD3	4	14	14	14	51.7	39.594	0	323.31
P42167;G5E972;P42167-3;P42167-2;P42166;H0YJH7	Lamina-associated polypeptide 2, isoforms beta/gamma;Thymopoietin;Thymopentin;Lamina-associated polypeptide 2, isoform	TMPO	6	8	8	8	32.2	50.67	0	25.498
O60784-4;O60784;O60784-2;V9GZ68;V9GYF4;B0QY02;Q6UW50;B0QY01;O60	Target of Myb protein 1	TOM1	9	2	2	2	8.5	50.268	0	4.577
Q9NS69	Mitochondrial import receptor subunit TOM22 homolog	TOMM22	1	1	1	1	8.5	15.521	0	17.115
P11387	DNA topoisomerase 1	TOP1	1	3	3	3	6.8	90.725	0	6.8152
P11388;P11388-2;P11388-3;P11388-4	DNA topoisomerase 2-alpha	TOP2A	8	10	10	10	8.6	174.38	0	18.069
J3KP33;P04637-5;P04637-6;P04637-2;P04637-3;P04637-4;P04637;E7ESS1;E7EQX7;A0A087WZU8;P04637-8;P04637-9;P04637-	Cellular tumor antigen p53	TP53	18	4	4	4	18.4	37.933	0	11.205
A0A087WWU8;Q5HYB6;P06753-4;P06753-3;P06753-6;P06753-5;P06753-2;D6R904;Q5VU61	Tropomyosin alpha-3 chain	TPM3;DKFZp686J1372	10	4	4	2	19.8	26.42	0	13.804
P67936	Tropomyosin alpha-4 chain	TPM4	1	2	1	1	9.7	28.521	0	5.5853
Q13595-2;Q13595-4;Q13595-3;Q13595	Transformer-2 protein homolog alpha	TRA2A	4	2	2	2	20.4	12.951	0	4.8781
P62995-3;P62995;H7BXF3;H7C2L4	Transformer-2 protein homolog beta	TRA2B	4	4	4	4	20.7	21.935	0	11.929
P19474;P19474-2	E3 ubiquitin-protein ligase TRIM21	TRIM21	3	11	11	11	33.5	54.169	0	272.28
C9JBA2;C9J974		TTC14	2	1	1	1	12	15.096	0.008606	1.8754
P68363;P68363-2;P68366-2;P68366	Tubulin alpha-1B chain;Tubulin alpha-4A chain	TUBA1B;TUBA4A	5	20	20	1	69	50.151	0	323.31
F5H5D3;Q9BQE3;F8VVB9	Tubulin alpha-1C chain	TUBA1C;TUBA1B	13	20	3	2	59.9	57.73	0	12.263
Q5JP53;P07437;Q5ST81;Q9BVA1;Q13885	Tubulin beta chain;Tubulin beta-2B chain;Tubulin beta-2A chain	TUBB;TUBB2B;TUBB2A	15	17	17	4	59.9	47.766	0	323.31
A0A0B4J269;Q13509;Q13509-2	Tubulin beta-3 chain	TUBB3	3	10	2	1	16.7	88.381	0	4.0223
P68371;P04350	Tubulin beta-4B chain;Tubulin beta-4A chain	TUBB4B;TUBB4A	13	17	5	3	57.3	49.83	0	296.52
Q9BUF5;K7ESM5	Tubulin beta-6 chain	TUBB6	2	6	1	1	17.3	49.857	0	17.14
Q3ZCM7;A0A075B736;Q5SQY0	Tubulin beta-8 chain	TUBB8	3	7	2	2	20.5	49.775	0	162.18
P49411	Elongation factor Tu, mitochondrial	TUFM	1	5	5	5	17.5	49.541	0	11.579
P10599-2;P10599	Thioredoxin	TXN	2	1	1	1	15.3	9.4519	0.007117	2.1178
Q01081-2;Q01081;K7EJH3;K7EJM7;M0QYK5;M0R2N4;Q01	Splicing factor U2AF 35 kDa subunit;Splicing factor U2AF 26 kDa subunit	U2AF1;U2AF1L4	11	3	3	3	13.3	27.882	0	4.4486
K7ENG2;P26368-2;P26368	Splicing factor U2AF 65 kDa subunit	U2AF2	3	2	2	2	11.1	33.901	0	2.6194
O15042-2;O15042;E7ET15;C9JB80;O15042-3	U2 snRNP-associated SURP motif-containing protein	U2SURP	11	20	20	20	25.6	118.23	0	237.49
E9PKP7;P17480-2;P17480;E9PLT2	Nucleolar transcription factor 1	UBTF	4	2	2	2	3.8	87.435	0	3.6576
Q9NYU2-2;Q9NYU2	UDP-glucose:glycoprotein glucosyltransferase 1	UGGT1	4	15	15	15	14.5	174.97	0	53.864
Q96IX5	Up-regulated during skeletal muscle growth protein 5	USMG5	1	2	2	2	43.1	6.4575	0	46.117
Q9Y5J1;J3QLD6;J3QRG0;J3QR85;J3KSR7	U3 small nucleolar RNA-associated protein 18 homolog	UTP18	5	2	2	2	5.2	62.003	0	3.4099
Q5H8X8;O95399;O95399-2	Urotensin-2	UTS2	3	1	1	1	5.8	16.276	0.008347	1.7355
Q9P0L0;Q9P0L0-2	Vesicle-associated membrane protein-associated protein	VAPA	3	4	4	3	18.9	27.893	0	7.9199
O95292;E5RK64;O95292-2	Vesicle-associated membrane protein-associated protein	VAPB	3	4	3	3	22.6	27.228	0	4.2484
A0A0A0MR02;P45880-2;P45880;P45880-1	Voltage-dependent anion-selective channel protein 2	VDAC2	7	4	4	4	26.2	30.348	0	46.65
P08670;B0YJC4	Vimentin	VIM	17	39	39	36	71.2	53.651	0	323.31
Q9C0J8	pre-mRNA 3 end processing protein WDR33	WDR33	4	3	3	3	3.2	145.89	0	6.6641
A0A087X295;Q9NNW5;E9PDU5;E9PBK6	WD repeat-containing protein 6	WDR6	5	14	14	14	20.6	125	0	49.253
Q9BQA1;H0Y711;Q9BQA1-2	Methylosome protein 50	WDR77	3	3	3	3	23.4	36.724	0	17.091
Q15007;A0A087X1R4;Q15007-2	Pre-mRNA-splicing regulator WTAP	WTAP	3	4	4	4	11.9	44.243	0	9.8002
P13010;C9JZ81	X-ray repair cross-complementing protein 5	XRCC5	2	3	3	3	9.6	82.704	0	21.655
P12956;B1AHC9;P12956-2	X-ray repair cross-complementing protein 6	XRCC6	3	7	7	7	16.7	69.842	0	14.463
P67809;A0A087X1S2;H0Y449	Nuclease-sensitive element-binding protein 1	YBX1	4	7	7	5	56.2	35.924	0	212.99
P16989-2;P16989-3;P16989;Q9Y2T7	Y-box-binding protein 3;Y-box-binding protein 2	YBX3;YBX2	4	4	2	2	34	31.947	0.007207	2.1669
P31946-2;P31946	14-3-3 protein beta/alpha;14-3-3 protein beta/alpha, N-terminally processed	YWHAB	2	3	1	1	12.3	27.85	0	3.1668
P62258;P62258-2	14-3-3 protein epsilon	YWHAE	7	13	13	11	57.6	29.174	0	137.89
P61981	14-3-3 protein gamma;14-3-3 protein gamma, N-	YWHAG	1	3	1	1	13	28.302	0	4.1691
Q04917	14-3-3 protein eta	YWHAH	1	3	1	1	13	28.218	0	4.0327
P27348;E9PG15	14-3-3 protein theta	YWHAQ	7	10	8	8	43.3	27.764	0	323.31
P63104;E7EX29;E7ESK7;B0AZS6;P63104-2	14-3-3 protein zeta/delta	YWHAZ	11	6	4	4	30.2	27.745	0	90.869
O75152;E9PQ61	Zinc finger CCCH domain-containing protein 11A	ZC3H11A	3	5	5	5	8.8	89.13	0	16.946
Q5T200-2;Q5T200	Zinc finger CCCH domain-containing protein 13	ZC3H13	2	2	2	2	1.5	184.87	0.00939	1.4419
G3V256;Q6PJT7-4;Q6PJT7-3;Q6PJT7-5;Q6PJT7-9;Q6PJT7-	Zinc finger CCCH domain-containing protein 14	ZC3H14	17	3	3	3	8.1	68.349	0	5.8988
H3BRH3;E7ERS3;Q86VM9	Zinc finger CCCH domain-containing protein 18	ZC3H18	3	1	1	1	12.8	21.008	0.009346	1.4294
A0A087WYV3;E7EPF0;A0A087WXF8;Q9NP64-2;Q9NP64-3;Q9NP64;A0A087WXU5	Nucleolar protein of 40 kDa	ZCCHC17	7	3	3	3	22.9	20.451	0	16.528
Q9NUD5	Zinc finger CCHC domain-containing protein 3	ZCCHC3	1	1	1	1	3.2	43.618	0.007286	2.216
H7C0K4		ZCWPW2	1	1	1	1	6.8	21.771	0.007987	1.522
Q5BKZ1;A0A0A0MRN4;Q5BKZ1-3	DBIRD complex subunit ZNF326	ZNF326	3	2	2	2	5	65.653	0	3.176
Q8IXQ4-2			1	1	1	1	8.9	22.226	0	2.6659
