# Supplementary material for: Extracellular calcium alters calcium-sensing receptor network integrating intracellular calcium-signaling and related key pathway
Source: Sci Rep. 2021 Oct 18;11:20576. doi: 10.1038/s41598-021-00067-2 (PMC8523568; doi:10.1038/s41598-021-00067-2)
Supplement: Supplementary file 3 — Supplementary Information 3. [file 41598_2021_67_MOESM3_ESM.docx]

Extracellular calcium alters calcium-sensing receptor network integrating intracellular calcium-signaling and related key pathways

Rakshya Gorkhali^1^, Li Tian^1^, Bin Dong^1^, Pritha Bagchi^3^, Xiaonan Deng^1^, Shrikant Pawar^2^, Duc Duong^4^, Ning Fang^1^, Nicholas Seyfried^4^, and Jenny Yang^1*^

**Affiliations:**

^1^Department of Chemistry, ^2^Department of Biology, Center of Diagnostics and Therapeutics, Advanced Translational Imaging Facility, Georgia State University, Atlanta, GA, 30303, USA

^3^Emory Integrated Proteomics Core, ^4^Department of Biochemistry, Emory University School of Medicine, Atlanta, GA, 30322, USA

*Corresponding author. Email: [jenny@gsu.edu](mailto:jenny@gsu.edu) (JJY)

**SUPPLEMENTAL FIGURES**

**Figure S1. The CaSR mediated intracellular Ca^2+^ signaling.** CaSR mediate intracellular signaling transduced through heterotrimeric G-proteins, Gq/11, Gi/o, G12/13, and Gs. Through Gq/11, the CaSR activates phospholipase C and mobilizes Ca^2+^_ER_ into cytosolic Ca^2+^ (Ca^2+^_cyt_). The rise in Ca^2+^_cyt_ communicates with Ca^2+^ stores, the ER, mitochondria and Golgi, via channels (in blue).

**Figure S2. The overview of the experimental workflow.** CaSR interacting proteins characterized using co-immunoprecipitation and MS detection. HEK293 cells transfected with FLAG-tagged CaSR pcDNA3.1 (positive control) or empty pcDNA3.1 (negative control) were subjected with 4mM Ca^2+^_ex_ and 2mM EGTA. The proteins identified using LC-MS/MS that were highly enriched in positive control by ≥ 2-folds were assessed as putative CaSR interacting proteins. For functional enrichment study, the complete list of CaSR interacting proteins were analyzed using the DAVID database. Further, the interaction between each protein was obtained using STRING PPI database search.

**Figure S3. MS on total cellular lysate on 106 proteins confirm no change in expression levels between treatments**. Total cellular protein expressions remain constant for the 106 putative CaSR interactors after the Ca^2+^ or EGTA treatments in cells transfected with FLAG-CaSR. Log_2_ intensity (Ca^2+^/EGTA) for total cell lysate after the treatments. Minimal change is observed between the treatment groups. Seven were not detected in the total lysates (MT-ATP8, WDR6, HBB, TBL2, RALBP1 and GPALPP1), two (PTPN1, TMCO1) had large errors between replicates, and four (HSPA6/7, IRS4, PTDSS1, SPTLC1) had greater than 1-fold changes.

**Figure S4*.* Scatter plot comparing pixel intensities in different channels for correlation analysis of CaSR (green), VAPA (red) and ER (magenta; calreticulin).** The scatter plots were compared for two conditions, physiological condition (DMEM 2.2 mM) and 4 mM Ca^2+^ treatment for 2 h. ρ represents Pearson’s coefficient.

**Figure S5. Scatter plot comparing pixel intensities in different channels for correlation analysis of CaSR (green), GRP78 (red) and ER (magenta; calreticulin)**. The scatter plot for two conditions were compared, physiological condition and 4 mM Ca^2+^ treatment for 2 h. ρ represents Pearson’s coefficient.

A

B

C

**Figure S6. Colocalization of CaSR with GRP78, STUB1, 14-3-3 η and VAPA with high Pearson’s coefficient in HEK293 cells. A.** Overexpressed CaSR co-localized with endogenous GRP78, STUB1 (CHIP), 14-3-3 eta and VAPA in HEK293 cells. Yellow represents co-localization. Scale bar; 5µm. **B.** Surface plot of pixel intensities analysis from confocal images of the area marked in grey square of HEK293 cells (in A) expressing FLAG-CaSR and endogenous proteins after 4mM Ca^2+^ treatment. **C.** Pearson’s coefficient of colocalization of CaSR with each of the interactors.

**Supplemental table 2. The functional annotation cluster generated through gene ontology for Ca^2+^ enriched CaSR interactors**

**Figure S7. Full gels for Fig 1A.** Co-immunoprecipitation experiments using anti-FLAG antibody on total cell extracts from HEK293 followed by western blot analysis with anti-CaSR antibody **(A)** and anti-GAPDH **(B)** (for total) was carried out in triplicates (I, II and III). Red blocks denote the regions of the blot from the main figures in replicate I and represent the protein blot of interest. Blue blocks represent the protein blots of interest in replicates II and III.

**Figure S8. Full gels for Fig 4A.** Co-immunoprecipitation experiments with either Ca^2+^ or EGTA treatments using anti-FLAG antibody on total cell extracts from HEK293 transfected with FLAG-CaSR and VAPA pcDNA3.1 followed by western blot on 50 µg total extract. Analysis with anti-CaSR antibody (**B and E) and** anti-VAPA antibody (**A and D**)**,** or anti-GAPDH (as control, **C**), was carried out in triplicate (I, II and III). Red blocks denote the regions of the blot from the main figures in replicate I and represent the protein blot of interest. Blue blocks represent the protein blots of interest in replicates II and III.

**Figure S9. Full gels for Fig 4B.** Co-immunoprecipitation experiments with either Ca^2+^ or EGTA treatments using anti-FLAG antibody on total cell extracts from HEK293 transfected with FLAG-CaSR and GRP78 pcDNA3.1 followed by western blot on 50 µg total extract. Analysis with anti-CaSR antibody (**B and E) and** anti-GRP78 antibody (**A and D**)**,** or anti-GAPDH (as control, **C**), was carried out in triplicate (I, II and III). Red blocks denote the regions of the blot from the main figures in replicate I and represent the protein blot of interest. Blue blocks represent the protein blots of interest in replicates II and III.
